# Structure-Based Virtual Screening of *Pseudomonas aeruginosa* LpxA Inhibitors using Pharmacophore-Based Approach

**DOI:** 10.3390/biom10020266

**Published:** 2020-02-10

**Authors:** Baki Vijaya Bhaskar, Tirumalasetty Muni Chandra Babu, Aluru Rammohan, Gui Yu Zheng, Grigory V. Zyryanov, Wei Gu

**Affiliations:** 1 Department of Pathophysiology, The Key Immunopathology Laboratory of Guangdong Province, Shantou University Medical College, Guangdong 515031, China; guiyu3210@gmail.com; 2 Department of physiology, Shantou University Medical College, Guangdong 515031, China; tmunichandrababu@gmail.com; 3 Department of organic and biomolecular chemistry, Ural Federal University, Yekaterinburg 620002, Russia; rammohan4ever@gmail.com (A.R.); gvzyryanov@gmail.com (G.V.Z.)

**Keywords:** PaLpxA, docking, pharmacophore, virtual screening, GBVI, ADME

## Abstract

Multidrug resistance in *Pseudomonas aeruginosa* is a noticeable and ongoing major obstacle for inhibitor design. In *P. aeruginosa,* uridine diphosphate N-acetylglucosamine (UDP-GlcNAc) acetyltransferase (PaLpxA) is an essential enzyme of lipid A biosynthesis and an attractive drug target. PaLpxA is a homotrimer, and the binding pocket for its substrate, UDP-GlcNAc, is positioned between the monomer A–monomer B interface. The uracil moiety binds at one monomer A, the GlcNAc moiety binds at another monomer B, and a diphosphate form bonds with both monomers. The catalytic residues are conserved and display a similar catalytic mechanism across orthologs, but some distinctions exist between pocket sizes, residue differences, substrate positioning and specificity. The analysis of diversified pockets, volumes, and ligand positions was determined between orthologues that could aid in selective inhibitor development. Thenceforth, a complex-based pharmacophore model was generated and subjected to virtual screening to identify compounds with similar pharmacophoric properties. Docking and general Born-volume integral (GBVI) studies demonstrated 10 best lead compounds with selective inhibition properties with essential residues in the pocket. For biological access, these scaffolds complied with the Lipinski rule, no toxicity and drug likeness properties, and were considered as lead compounds. Hence, these scaffolds could be helpful for the development of potential selective PaLpxA inhibitors.

## 1. Introduction

Multidrug-resistant (MDR) bacteria have been recently denoted ESKAPE 2242 (Enterococcus faecium, Staphylococcus aureus, Klebsiella pneumoniae, Acinetobacter baumannii, Pseudomonas aeruginosa and Enterobacter) pathogens [1]. P. aeruginosa is an opportunistic MDR pathogen, with infection mostly occurring in humans during hospitalization. Since severe infection can be life-threatening, researchers have attempted to design novel therapeutics [2]. Despite the fact that current antimicrobials impede essential metabolic pathways in bacteria, they have become less effective due to the emergence of drug resistance [3,4]. These obstacles can be overcome with the development of novel therapeutics against alternative or drug-resistant targets. In P. aeruginosa, lipopolysaccharide (LPS) is a vital part of the outer membrane, as in most Gram-negative bacteria, that protects it from its environment and acts as a major virulence factor. LPS is a complex glycolipid mainly composed of three structurally different parts: (1) Lipid A, which anchors LPS to the outer membrane; (2) a core oligosaccharide; and (3) O-antigen repeats, which are exhibited on the bacterial cell surface [5,6,7]. Lipid A biosynthesis is an evolutionarily conserved metabolic pathway among Gram-negative bacteria requiring a series of nine enzymes, including LpxA, LpxC, LpxD, LpxH, LpxB, LpxK, WaaA, LpxL and LpxM. Inhibition of the first six sequential enzymes inevitably kills the bacteria [8,9], while no mammalian counterparts exist. Therefore, these enzymes become promising targets for the development of potent antibacterial drugs.

A series of reactions are involved in lipid A biosynthesis. The initial three reactions are carried out by LpxA, LpxC and LpxD. In the first reaction, the 3OH of UDP-GlcNAc are acylated with a β-hydroxymyristoyl moiety from the acyl carrier protein (ACP) by LpxA, which is thermodynamically unfavorable [10,11]. The subsequent reactions are thermodynamically favorable and catalyzed by enzymes. LpxA is a functional homotrimer, with each monomer exhibiting a left-handed parallel β-helix fold covered with 24 complete and six partial specific hexad repeats, with every turn in the β-helix formed by a single hexapeptide [12]. LpxA has the ability to detect the specific length of the acyl hydrocarbon through a hydrophobic cleft, either long or short. However, these acyl hydrocarbon residues are implicated in the identification of a specific length of acyl chains, a so-called hydrocarbon ruler, which is different across species [13]. Recently, the crystal structure of PaLpxA has been resolved with substrate and product, providing definite enzyme catalysis and substrate-binding evidence that aid in the development of new classes of antibacterial agents [14]. PaLpxA complexed with substrate (UDP-GlcNAc) and product revealed UDP-GlcNAc to be bound at the catalytic cleft of the protein–protein interface. UDP interacts with the UDP pocket residues of Ile148, Phe166, Asn194, Glu196, Arg200 and Arg201 of one monomer, whereas GlcNAc interacts with the GlcNAc pocket residues of Asp70, Leu71, His121, His140, His156 and Gln157 from another monomer connected by a diphosphate bridge [14,15]. Mutagenesis studies documented His121 at the GlcNAc pocket participates in catalysis to deprotonate the UDP-GlcNAc and facilitate a nucleophilic environment for the attachment of the acyl moiety [13]. So far, some peptide and small molecule inhibitors have been reported to thwart LpxA function by competing with acyl-ACP and UDP-GlcNAc in various orthologues [16]. Peptide920 is a peptide inhibitor 15 amino acids long competing for acyl chain binding site with inhibitory concentration of *K*i = 50 nM against *Escherichia coli* LpxA [17]. RJPXD33 is an antimicrobial peptide which showed dual inhibition for LpxA and LpxD by competing with acyl-ACP substrate [18]. Recently, peptideCR20 was reported with IC_50_ of ≈50 nM against *E. coli* LpxA [19]. Even though these peptides exert potential activity, they confer poor bioavailability and susceptibility. Alternatively, small molecules with substrate-mimicking properties have been discovered for *Moraxella catarrhalis* [20]. However, specific inhibitors have not been investigated for PaLpxA and must be explored for persuasive inhibitors to thwart the *P. aeruginosa* infections. In this scenario, our efforts are utilized to develop effective PaLpxA inhibitors using predictive in silico experiments and to manage the clinical settings for effective management of infectious diseases. 

## 2. Materials and Methods

### 2.1. Binding Pocket and Volumetric Analysis

LpxA crystal structures—without water, cofactors and cocrystal ligands—of *P. aeruginosa* (PDB ID: 5DEP, 5DEM, 5DG3), *A. baumannii* (PDB ID:4E6Q) [21], *E. coli* (PDB ID:2JF3), *Helicobacter pylori* (PDB ID: 1J2Z) [22], *Leptospira interrogans* (PDB ID: 3HSQ) [23] and *Burkholderia thailandensis* (PDB ID:4EQY) [24] were retrieved from the Protein Data Bank (PDB). All crystal structures were subjected to root mean square deviation (RMSD) analysis, binding cavity volumetric and shape analysis carried out using the Site Finder module of the molecular operating environment (MOE) program [25]. Site Finder calculates possible active sites in the receptor using 3D atomic coordinates. The site finder parameters were set as follows: Probe radius 1 was 1.4 Å, probe radius 2 was 1.8 Å, isolated donors/acceptors were 3, connection distance was 2.5 Å, minimum site size was 3 Å, and radius was 2 Å. This module uses the geometric category of methods and is primarily based upon the alpha spheres, which are generalized convex hulls [26]. The tight atomic packing regions were identified and filtered out for being over-exposed to solvent. Then, the site was classified as either hydrophobic or hydrophilic. The collected alpha spheres were clustered by using a double-linkage algorithm to produce ligand-binding sites and rank the sites according to their propensity for ligand binding (PLB) based on the amino acid composition of the pocket [27].

### 2.2. Ligand Preparation

The NCI drug database contains 265,242 heterogenous compounds, including 3D atomic coordinates, molecular formulas, molecular weights, and IUPAC structure identifiers, such as standard InChI and standard InChIKey, all of which were downloaded from the National Cancer Institute (http://cactus.nci.nih.gov/download/nci). This dataset was launched into MOE through database viewer and primarily subjected to wash to correct errors in the structures, such as single bonds, protonation, disordered bond lengths, tautomers, ionization states, and explicit counter ions. All the compounds were converted to 3D conformations, hydrogen and atomic partial charges were applied, and energy minimization was performed with an MMFF94x force field for small molecules. The refined dataset was utilized for further experiments.

### 2.3. Pharmacophore Modeling and Virtual Screening

The complex-based pharmacophore technique was used to improve the drug development process. A pharmacophore is the combined steric and electronic features of the ligand that are necessary to ensure the optimal supramolecular interactions with a specific biological target and to inhibit its biological actions. It emphasizes the characteristic that various chemical moieties might share a similar property and so be characterized by the same feature. In MOE, an inbuilt module pharmacophore query creates a set of query features from annotation points of the ligand, receptor and ligand complex, and receptor only. These features explain the crucial atoms and groups, namely, hydrogen donors, hydrogen acceptors, aromatic centers, R-groups, charged groups and bioisosteres. Therefore, in the current study, combined complex-based or receptor-based pharmacophore modeling was used to identify salient features and create a pharmacophore query to screen virtual compound libraries for novel PaLpxA inhibitors. Thus, a 3D pharmacophoric features query of the UDP-GlcNAc pocket of PaLpxA was generated using the least square (LS) program of the pharmacophore query editor of MOE. The query consisted of a set of constraints on the location and type of pharmacophoric features. The force field parameters were set up using the potential setup in the MOE as follows: The force field was set to amber10:EHT [28]; solvation was set to R-field and bonded, van der Waals, electrostatics and restrains were enabled. Hydrogen and partial charges were adjusted. Subsequently, in the LigX panel, the receptor strength was tethered to 5000 to keep the receptor rigid. This enabled 3D protonation and deleted the water molecules farther than 4.5 Å from the receptor or ligand. Consequently, the pharmacophore query was created by applying the following parameters: The scheme was set to EHT to define the set of attributes that are used to construct the ligand annotation points. These annotation points were represented in the form of different colors—green for hydrophobic, purple for hydrogen bond donor, cyan for hydrogen bond acceptor and orange for aromatic. The pharmacophore query was validated by performing the pharmacophoric features search and the query was refined by changing features iteratively. For potential leads, pharmacophoric features were condensed with reference to the key residues within the binding pocket for better binding and orientation of the leads. A virtual screening (VS) campaign was used as an *in silico* sieve using the modified and validated pharmacophore model to screen the refined NCI virtual compound library [29,30]. The lowest binding energy conformations for each ligand were saved in a separate MOE database by using the conformation import methodology. Thereafter, each compound conformer was filtered by the pharmacophore model based on satisfying the marked pharmacophoric features to be considered as a virtual hit. Subsequently, the identified virtual hits were plotted on the designed pharmacophore and allowed for docking studies against PaLpxA.

### 2.4. Molecular Docking

Molecular docking was performed for the pharmacophoric hits using the docking module of MOE [31]. Hydrogens were added to the protein and subjected to protonation 3D. Partial charges were added using the method of AMBER99 and energy was minimized using the following parameters and methods. The gradient was set to 0.00001, and the MMFF94x force field was used with an enable cut-off value set from 8 to 10; solvation was set to distance mode; dielectric was set to 1; exterior was set to 8; and partial charges were fixed for required calculations. After minimization, the stabilized protein molecule was used for further docking analysis. The docking site was set to the ligand atoms within the crystal structure of PaLpxA. The descriptor filter was specified to satisfy the Lipinski rule of five. The docking parameters were set as follows: The placement methodology was set to triangle matcher to calculate initial poses and scores. The rescoring methodology was set to the London dG function that ranks the docked poses. The maximum number of poses retained the 30 lowest docking poses and removed the duplicates. The RMSD was calculated within 2 Å between the docked pose and the crystal pose and considered as reliable docking for reproducibility. After docking, the best lead molecules were identified based on the maximum docking score, and binding angles and bond lengths were visually inspected using LigPlot and PyMOL.

### 2.5. Born Interaction Energies and Binding Affinities

Born-volume integral (GB/VI) implicit solvent methods were implemented in MOE to determine the binding affinities of lead molecules [32]. Van Der Waals, Coulomb electrostatic interactions, and implicit solvent interactions were termed the Born interaction energy, which determined non-bonded interactions between the ligands and the protein. During the binding affinity assessment, the draining energies, and solvent molecules of the ligand and the protein were ignored. The London scoring method was represented in kcal/mol units for the binding affinity calculations. The binding pocket residues and ligands were kept flexible, whereas the binding pocket was kept rigid to tether restraints and discourage gross movements. Energy minimization for the protein–ligand complex was employed, and the binding affinity was calculated.

### 2.6. Protein–Peptide Docking

Protein–protein docking was accomplished using the HDOCK server [33]. The hybrid docking protocol uses a fast Fourier transform (FFT) scoring method by this server [34]. PaLpxA, *E. coli* LpxA, peptide920 and RJPXD33, when subjected to docking, showed 100 binding modes with binding energies. Of these, the top 10 ranked models were selected and manually inspected to ascertain the proper binding conformation overlaid with reference crystals structures.

### 2.7. Bioavailability

Molecular properties, the Lipinski rule of five (molecular weight < 500, H-bond acceptor < 10, H-bond donor < 5, and cLogP < 5), drug likeness, and toxicity properties (mutagenic, tumorigenic, irritant and reproductive effects) were predicted using ADME (absorption, distribution, metabolism and excretion) properties and assessed using the Swiss ADME (Lausanne, Switzerland) and FAF-Drugs3 servers (Paris, France) [35,36].3. Results and Discussion

## 3. Results and Discussion

### 3.1. PaLpxA-Substrate Analysis

**Figure 1:**
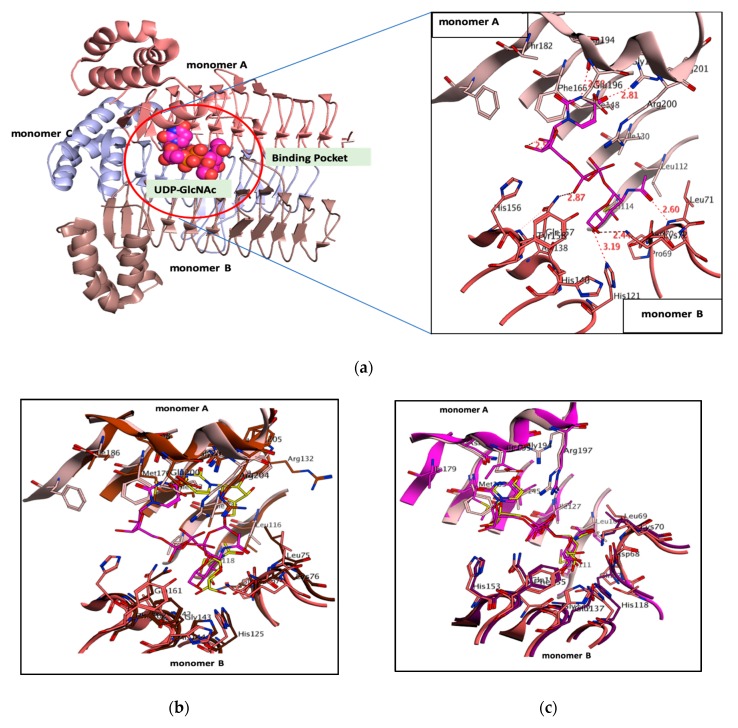
(**a**) PaLpxA consists of a homotrimer and each monomer is labeled with different color, UDP-GlcNAc is positioned within the diversified active site that is marked with a red circle between monomer A and B. LpxA is shown in the cartoon, whereas UDP-GlcNAc is shown in space-filling model. 3D binding interaction of UDP-GlcNAc (magenta) with key residues of PaLpxA pocket (light salmon for monomer A and dark salmon for monomer B). (**b**) Overlay of UDP-GlcNAc (magenta) within the diversified pocket of PaLpxA with UDP-GlcNAc (yellow) within the diversified pockets of *E. coli* LpxA dimer (dark brown for monomer A and light brown for monomer B) and (**c**). UDP-GlcNAc (yellow) of *B. fragilis* LpxA dimer (light magenta for monomer A and dark magenta for monomer B)**.** Hydrogen bonds are represented as dashed lines in cyan and green colors. Binding interactions are shown in blue and green colors.

Biologically relevant functional homotrimer (monomer A, B and C) PaLpxA crystal structures with the apo form, complexed with respective substrate and product, UDP-GlcNAc and UDP-3-(R-3-hudroxydecanoyl)-GlcNAc, were resolved at 1.8 Å and were available in the PDB. PaLpxA monomers A, B and C were identical, with some structural distinction within the loop region L1 and L2. PaLpxA monomer consisted of two discrete domains, the N-terminal β-strand domain and C-terminal α-helical domain. The catalytic site lay between the protein–protein interface where the C6 and C9 coils meet from adjacent monomers A and B. In the PaLpxA-substrate complex, the binding position of UDP-GlcNAc was placed at the monomer A–monomer B interface, where UDP was situated at monomer A, namely the UDP pocket, and GlcNAc was pinpointed at another monomer B in the GlcNAc pocket; the diphosphate group acted as a connecting bridge (Figure 1a). UDP-GlcNAc formed key contacts with active pocket residues, and the uracil moiety formed critical interactions, such as O atom hydrogen bonds with Arg201, N atom hydrogen bonds with Asn194, and a Pi–Pi interaction between the uracil ring and aromatic ring of Phe166 from monomer A. In addition, some van der Waals forces resulted from the Ile148 and Glu196 towards the uracil ring. The 3OH of the ribose sugar displayed an interaction with His156 of monomer B. The diphosphate group conferred strong interactions with both monomers A and B, with Arg200 from monomer A forming two strong hydrogen bonds with the O atom of the α-phosphate, and in turn, the O atom of the α-phosphate hydrogen bonded with Gln157 from monomer B and the O atom of the β-phosphate. GlcNAc solely formed contact with key residues of the GlcNAc pocket of monomer B. The acetyl group was located on the second position and hydrogen bonded with the backbone of Leu71 by donating an electron. The 3OH formed a strong polar bond with the catalytic base Nε2 of His121, allowing this residue to perform a nucleophilic attack by deprotonating the 3OH. The 4OH also conferred a weaker polar bond with the Nε2 of His121, and the 6OH formed two strong polar bonds with Lys72 and His140. Furthermore, the crystal structures of LpxA-substrate complex from two orthologues, *E. coli* and *B. fragilis*, were resolved and these were subjected to overlaying with the binding pose of UDP-GlcNAc of PaLpxA. Noteworthy, significant marked variation was perceived with the orientation of the uridine ring in both *E. coli* LpxA and PaLpxA (Figure 1b). The uridine ring, sugar and α-phosphate moieties were flipped away in the pocket, with no perturbations in the GlcNAc position in *E. coli* LpxA. The UDP moiety was unable to form a Pi–Pi interaction, due to the absence of a Phe residue, and formed a polar interaction with His156 by flipping. Moreover, PaLpxA UDP-GlcNAc showed an identical binding position with *B. fragilis* (Figure 1c). Owing to the variation in substrate orientation, the pocket residues played an important role in the specificity and position of the substrate across orthologs.

### 3.2. LpxA Pocket Analysis among Orthologs

Protein–ligand interactions are a fundamental phenomenon in all biological mechanisms; however, these binding interactions are certainly specific due to the distinct pocket and ligand properties. Exclusively, the binding pocket, shape, and residue physico-chemical properties determine the nature of the pocket and distinguish from orthologs. Accordingly, binding pocket analysis and comparison can shed light on selectivity and binding properties of the ligand, and most importantly, resolve the molecular mechanism between the protein and the ligand in biological systems. In this study, the PaLpxA structure was superimposed on LpxA orthologs from different bacterial pathogens to determine differences in the key residues and shape of the pockets. The RMSD was calculated between PaLpxA and different LpxA orthologs, and superimposition of PaLpxA revealed no significant structural deviations, showing 0.516 Å for *A. baumannii*, 0.628 Å for *E. coli*, 0.628 Å for *B. thailandensis*, 0.638 Å for *H. pylori* and 0.803 Å for *L. interrogans*.

**Figure 2:**
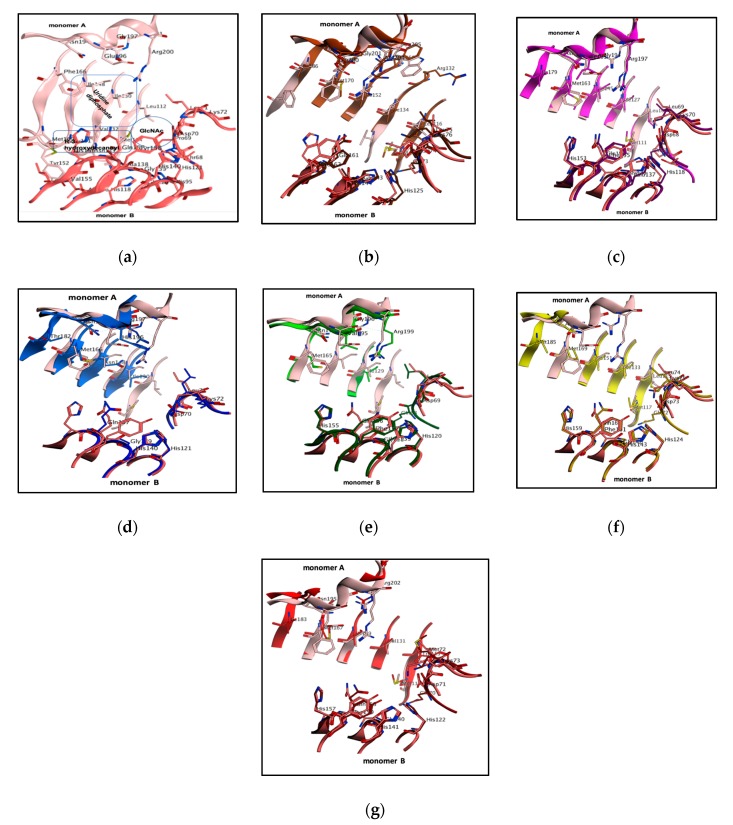
The structural orientation of UDP-GlcNAc binding cavity and superimposition of PaLpxA with six different bacterial LpxAs (b-g) to design marked inhibitors. (**a**) The binding orientation of UDP-GlcNAc within the bifurcated PaLpxA active pocket, with regions highlighted as a square region for UDP binding, a circle for GlcNAc binding and a rectangular region for product binding. LpxAs are depicted as cartoons and colored with different orthologues, essential pocket residues are shown as classical sticks and colored with elements. Overlays of PaLpxA (light salmon for monomer A and dark salmon for monomer B) with (**b**) *E. coli* dimer (dark brown for monomer A and light brown for monomer B), (**c**) *B. fragilis* dimer (light magenta for monomer A and dark magenta for monomer B), (**d**) *H. pylori* dimer (light blue for monomer A and dark blue for monomer B), (**e**) *L. interrogans* dimer (light green for monomer A and dark green for monomer B), (**f**) A. *baumannii* (light yellow for monomer A and dark yellow for monomer B), and (**g**) *B. thailandensis* (light brown for monomer A and dark brown for monomer B).

Furthermore, an assessment of the UDP-GlcNAc binding pocket revealed significant differences in the critical residue composition at both the uridine and GlcNAc binding sites, as shown in Table 1**.** The notable change at the uridine binding site of PaLpxA was that Phe166 was substituted for Met between orthologues, and a structural shift was noticed with the key residue, Arg202, from *A. baumannii* (Figure 2f). In the case of the GlcNAc pocket, the most prominent difference was that Tyr158 in *PaLpxA* mutated with Phe162, Phe161, Phe158, and Phe157 residues in *A. baumannii, E. coli, H. pylori* and *L. interrogans*, and Met72 and Phe72 were mutated in *B. thailandensis* and *L. interrogans* instead of Lys72. *B. thailandensis* had a smaller cavity with variations in the flexible loop region (L1) that consisted of Gln70 and Met72, instead of Lys72 and Leu71. *H. pylori* showed an outward conformation of Lys72 at the L2 loop region. Conversely, the catalytic His residue and pocket histidine residues were found to be highly conserved with slight conformational changes, excepting Tyr158. Significant variations were also observed in the flexible loop region.

**Figure 3:**
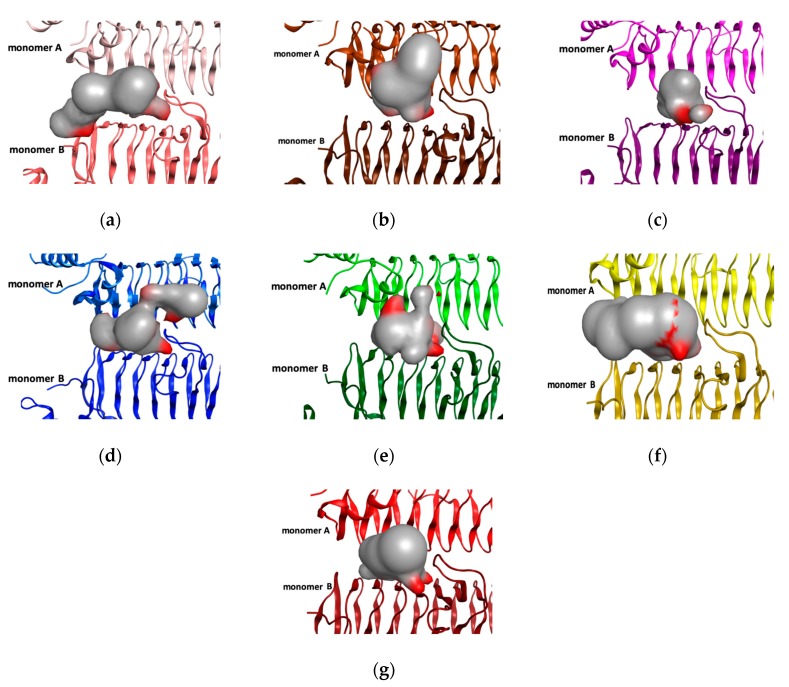
LpxA-UDP-GlcNAc binding cavity volume and shape analysis. The substrate-binding cavity is enveloped with a smooth surface between adjacent monomers A and B. The grey color represents the hydrophobic region and red color represents the hydrophilic region in different orthologues such as (**a**) *P. aeruginosa* LpxA dimer (light salmon for monomer A and dark salmon for monomer B), (**b**) *E. coli* LpxA dimer (dark brown for monomer A and light brown for monomer B), (**c**) *B. fragilis* LpxA dimer (light magenta for monomer A and dark magenta for monomer B), (**d**) *H. pylori* LpxA dimer (light blue for monomer A and dark blue for monomer B), (**e**) *L. interrogans* LpxA dimer (light green for monomer A and dark green for monomer B), (**f**) A. *baumannii* LpxA dimer (light yellow for monomer A and dark yellow for monomer B), and (**g**) *B. thailandensis* LpxA dimer (light brown for monomer A and dark brown for monomer B).

In addition, the binding pocket shape was an essential feature that determined the appropriate compounds with preferred biological targets. Several reports reinforced the molecular shape, which plays a crucial role in the biological activity. Volumetric binding cavity analysis was performed and is depicted in Figure 3. Pocket analysis exemplified different molecular pocket shapes with various volume sizes and residue compositions within the LpxA adjacent monomers; these are represented in the supplementary Table S1. The percentage of identity of the pocket residues between LpxA orthologues compared with PaLpxA that enlighten *B. fragilis* showed the highest identity of 58%, while *E. coli* and *B. thailandensis* showed 37%, *L. interrogans* showed 31%, and *H. pylori* and *A. baumannii* showed 28% and 18%, respectively (Supplementary Table S2). Moreover, volumetric binding for *P. aeruginosa*, *H. pylori* and *L. interrogans* showed the largest pocket surfaces with volume sizes of 224 Å, 209 Å, and 205 Å, respectively, while *B. fragilis* and *B. thailandensis* showed smaller pocket surfaces with volumes of 123 Å and 145 Å, respectively. *E. coli* and *A. baumannii* showed the most similar pocket surface volumes of 156 Å and 151 Å, respectively. The tendency of ligand binding residues in the pocket revealed 5.1 for *P. aeruginosa*, 3.7 for *H. pylori* and 5.2 for *L. interrogans*, 3.2 for *B. fragilis*, 4.2 for *B. thailandensis*, 3.9 for *E. coli* and 3.6 for *A. baumannii*. Intriguingly, the highest numbers of hydrophobic contact atoms, such as 52 and 53, were found in *P. aeruginosa* and *L. interrogans*, *H. pylori* had 44 atoms, and *E. coli* and *B. fragilis* had 36 and 32 atoms, respectively, whereas *A. baumannii* and *B. thailandensis* had fewer numbers of hydrophobic atoms, with 22 and 18, respectively. In addition, 113, 106 and 105 side chain atoms were implicated in the ligand-binding pocket from *P. aeruginosa*, *H. pylori* and *L. interrogans*, respectively. Different side chain atoms were involved in the pockets, such as 76 atoms in *E. coli*, 69 atoms in *B. fragilis*, 61 atoms in *A. baumannii* and 60 atoms in *B. thailandensis*. The study of shape and unique features of the pocket has gained importance in molecular pharmacology and the aforementioned results might be helpful in the design of specific LpxA inhibitors. 

### 3.3. PaLpxA-Peptide Docking 

**Figure 4:**
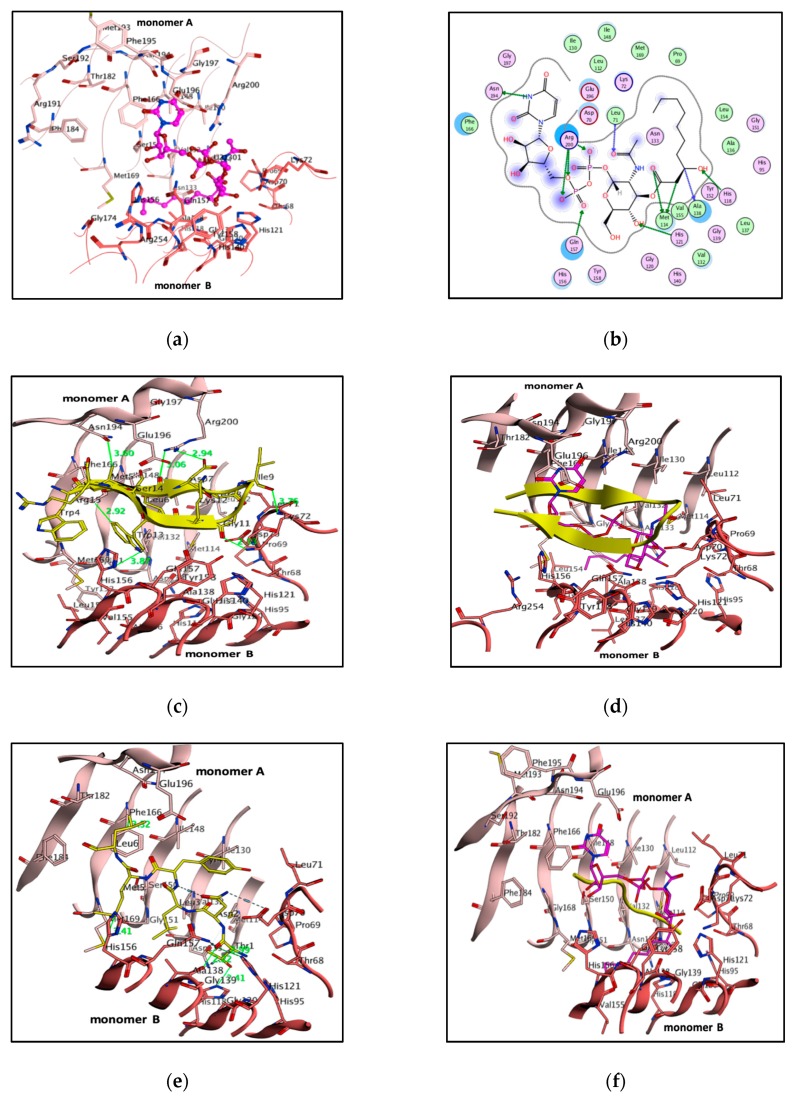
(**a**) 3D and (**b**) 2D representations of the PaLpxA-UDP-3-O-(R-3-hydroxydecanoyl)-GlcNAc product complex with binding interactions of key residues. PaLpxA dimer (light salmon for monomer A and dark salmon for monomer B) is depicted with wires and the product is rendered in balls and sticks and colored with magenta by the element, (**c**) Docked conformation of peptide920 (yellow) within the active site forms contacts with key residues of PaLpxA. (**d**) When overlaid with the peptide-product complex (magenta), the peptide is shown with β-hairpin folded sheets along with sticks by labeling. (**e**) Docked conformation of the RJDX33 peptide (yellow) inhibitor within active site showing contacts with key residues of PaLpxA. (**f**) When overlaid with the peptide-product complex (magenta), the RJDX33 peptide is rendered with loops in yellow. Binding interactions are represented in green color with distances (Å).

In the PaLpxA-UDP-3-O-(R-3-hydroxydecanoyl)-GlcNAc product complex, the acyl chain was linked with the 3O of GlcNAc and extended towards the hydrophobic acyl chain binding cavity (Figure 4a), wherein the acyl chain formed polar interactions by donating electrons to the side chain of Met114 of monomer A and backbone of Ala138, and accepting an electron from His118 of monomer B. Apart from this, numerous hydrophobic contacts were also observed with pocket surrounding residues: Val132, Asn133, Gly151, Tyr152 and Met169 from monomer A; and Ala136 and Leu154 from monomer B. Fascinatingly, functional aspects of product formation from previous reports showed the acylated product length could be restricted among orthologs in the hydrophobic tunnel, the so-called hydrocarbon ruler that designates specific residues that assess the suitable acylated chains, or else it would not permit binding due to several deficient molecular interactions. For instance, *E. coli* has residue His191 to act as a hydrocarbon ruler that allows only a 14C hydrocarbon chain, whereas *L. interrogans* has a Lys171 residue to allow a 12C hydrocarbon chain and *P. aeruginosa* has a Met169 that permits a 10C hydrocarbon chain. Stabilization of the transition state during acylation of UDP-GlcNAc occurred through a backbone amide group of a conserved Gly residue that acted as the oxyanion hole. 

Previous studies reported several competitive peptide inhibitors that target the acyl binding pocket of LpxA from *E. coli*. Williams et al. resolved the crystal structure of LpxA (1.8 Å) with the pepide920 peptide inhibitor (WMLDPLAGKWSR), and Jenkins et al. determined the crystal structure of LpxA (1.9 Å) in complex with the RJPXD33 (TNLYML) peptide inhibitor from *E. coli* at different resolutions [16,17,18]. These antimicrobial peptide inhibitors selectively compete with an acyl-ACP substrate and potentially inhibit *E. coli* LpxD and LpxA acyltransferases. However, in this study we conducted docking experiments with these peptides for PaLpxA using the HDOCK server, the results of which are presented in Table 2. Intriguingly, docking results showed peptide920 had the highest binding energy of –263 kcal/mol, which was higher than RJPXD33 (–177 kcal/mol), for PaLpxA, which was bound at the UDP-GlcNAc binding pocket. In the PaLpaA-Peptide920 complex, Peptide920 showed a β-hairpin folded conformation in the bifurcated active pocket, and N and C terminals exposed towards the solvent with the hairpin protruding into the pocket, as observed in the *E. coli* LpxA-peptide920 complex (Figure 4b). Peptide920 formed crucial interactions with substrate binding residues, N-terminal Met5 bonded with the uracil-binding residue Asn194, and Leu6 and Asp7 bonded with α-phosphate-binding residue Arg200 from monomer A. The hairpin loop consisted of four residues among Ile9 and Gly11 bonds, with Lys72 from monomer A and C-terminal Trp13 displaying two bonds, such as one polar bond with His156 from monomer B and a π–π bond with Phe166 from monomer A.

While the RJPXD33 peptide inhibitor tethered vertically up at the bifurcated pocket of PaLpxA, the Thr1, Asn2, Leu3 and Met5 residues of the peptide occupied the GlcNAc binding pocket and hydrophobic cleft of monomer B. Thr1 formed hydrogen bonds with catalytic residues His121, Gln157 and His118 of monomer B. Asn2 displayed one polar bond with Asp70, and Met5 directly interacted via an S–S bond with Met169, the acyl chain ruler. Leu6 and Tyr4 were positioned at the uracil binding site of monomer A. Leu6 interacted with Phe166 through a π–π interaction (Figure 4c). However, peptide inhibitors lack the peptide delivery system into the bacterial cytoplasm, and so it is not an optimistic approach for targeting cytoplasmic proteins. However, the peptide binding analysis can guide us to design safer and greater bioavailability inhibitors. An alternative approach is the design of small molecules that target the LPS biosynthesis pathway.

### 3.4. Pharmacophore-Based Virtual Screening

**Figure 5:**
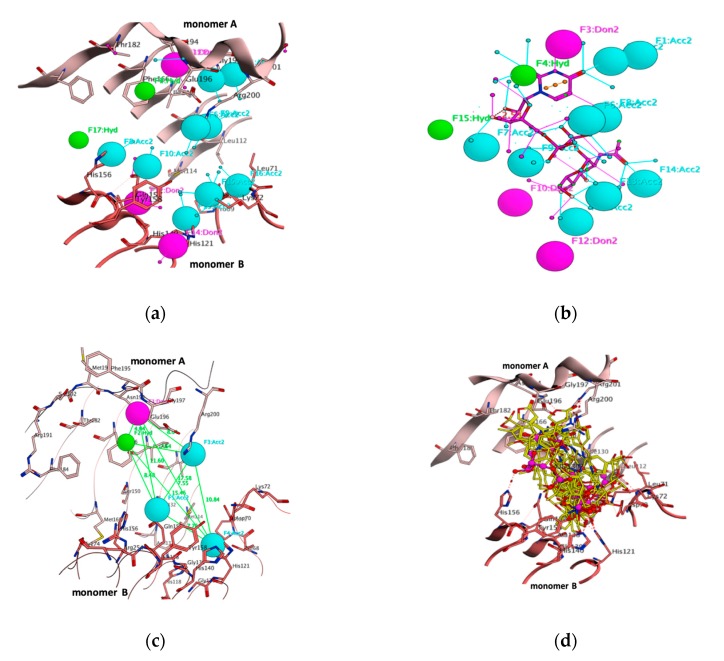
(**a**) Receptor 3D pharmacophore annotation of the UDP-GlcNAc pocket of PaLpxA (light salmon for monomer A and dark salmon for monomer B). (**b**) 3D pharmacophore annotation of UDP-GlcNAc with projections pinpoint the receptor pharmacophores. (**c**) Key pharmacophoric features with distance constraints within the pocket. (**d**) Superimposition of top-ranked pharmacophoric hits with the binding conformation of UDP-GlcNAc. When overlaid with the binding conformation of UDP-GlcNAc (balls and sticks, magenta), the virtual hits are rendered with sticks in yellow. (Don&Acc: Hydrogen bond donor/acceptor, Aro: Aromatic center, Don: Hydrogen bond donor, Acc: Hydrogen bond acceptor).

Designing effective and specific drugs depends mainly on the volume and shape of the target protein cavity combined with the physicochemical characteristics that are implicated in the binding and position of the ligand. Pharmacophore modeling is a persuasive process to determine novel hits and lead molecules. In this study, a pharmacophore model was developed using a complex-based approach. In this scenario, physicochemical characteristics, spatial arrangements, and the shape and volume of the binding cavity were considered. A receptor–ligand complex-based pharmacophore model was accomplished. A pharmacophore model constructed with fourteen pharmacophoric features, namely, three hydrogen bonding donors, nine hydrogen bond acceptors and two aromatic centers (Figure 5a,b). However, as aforementioned, the substrate and product shared pharmacophoric groups with binding pocket residues of PaLpxA. Three residues in the structure—Asn194, Phe166 and Arg200— in monomer A formed a hydrogen bond with the uracil moiety. Gln157 and His121 of monomer B formed bonds with GlcNAc and performed catalysis. Five pharmacophoric features—F3 (Donor), F4 (aromatic), F6 (acceptor), F12 (acceptor), and F9 (acceptor)—were identified as essential features that display critical interactions with substrate binding residues and the pharmacophore model was generated (Figure 5c). The developed 3D pharmacophore model provided an important functionality that contributed to the selection of selective inhibitors and, therefore, was used as a filter in the virtual screening process. The NCI database contains 265,242 heterogeneous compounds and was subjected to virtual screening against our developed pharmacophore model that deliberated 1718 virtual hits, which were superimposed with a query compound. Accordingly, these screened hits were docked and the top ranked lead molecules were identified, based on the best binding energies with corresponding binding conformation within the pocket, and overlaid with substrate to ensure conformation of the leads (Figure 5d).

### 3.5. Molecular Docking

To evaluate the lead molecules, all the pharmacophoric hits were docked in the GlcNAc binding pocket of LpxA by using the docking module of MOE. The docked conformation of ligands approving to the highest docking score with a low RMSD value was selected as the most efficient binding orientation. To validate the docking protocol, UDP-GlcNAc was redocked into the binding pocket, and the best binding pose, with a binding energy of –6.9 kcal/mol, was superimposed with crystal ligand that revealed a similar conformation with RMSD of 1.2 Å, indicating that the docking protocol was reliable (Figure S2a). The binding energy denotes the sum of the total internal energy, torsional free energy and intermolecular energy subtracted from the unbound energy system. The lowest free energy binding conformation specifies stable protein and ligand complex system anddetermine highest affinity among them. Docking results of virtual hits showed the binding energies ranged from –6.5 to –10.5 kcal/mol. A total of 137 top ranked ligands were selected based on the binding energies from –10.5 to –7.4 kcal/mol, with RMSD tolerance of 2.0 Å for further affinity evaluations. 

### 3.6. Born Interaction Energies and Binding Affinities

**Figure 6:**
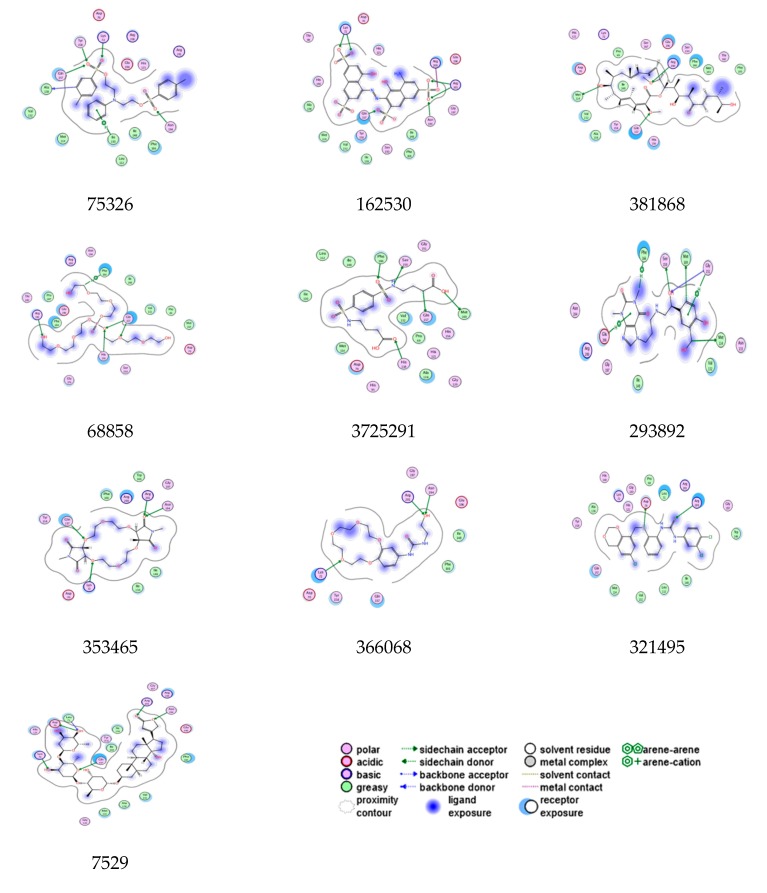
2D interactions of the top rank inhibitors with key residues of PaLpxA.

The generalized Born/volume integral (GB/VI), Born interaction energies and binding affinities were calculated using MOE to uncover the binding energy, binding affinity and interactions of lead molecules. The energy minimization employed for each complex and binding affinity (Kcal/mol) was measured. By analyzing the binding affinity and binding energy, 10 molecules could unambiguously be chosen from the top rank docked compounds (Table 3). Therefore, the protein–ligand interactions were analyzed to reveal significant implicated atoms with tether restraint through 2D assessments that exclude the false positives due to assumptions and deficiencies in the docking protocol and the scoring methods (Figure 6) [25,37].

**Figure 7:**
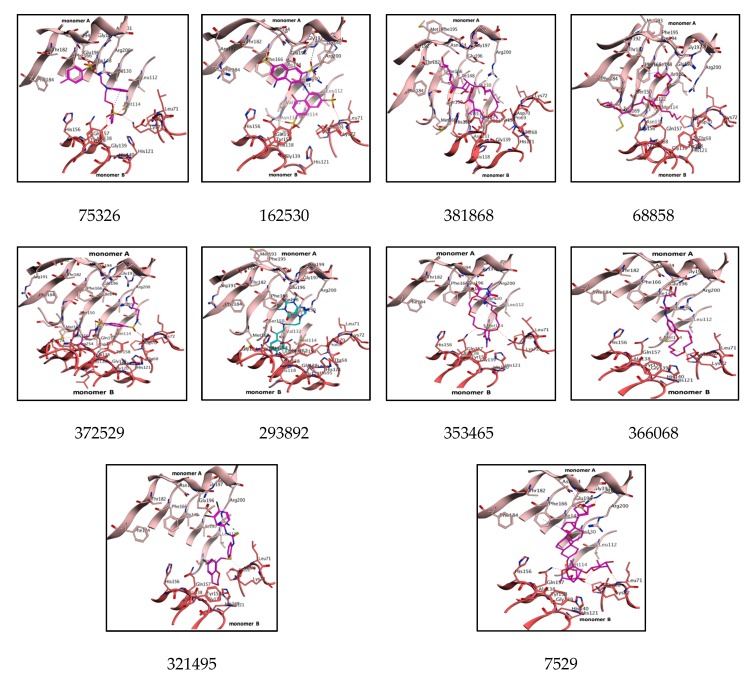
Top-ranked inhibitors (magenta) are rendered in the UDP-GlcNAc pocket and key residues are shown as sticks, colored by atoms and labeled. PaLpxA dimer (light salmon for monomer A and dark salmon for monomer B) is represented as a cartoon. Binding interactions are displayed by red dotted lines.

Compound 75326 formed six bonds with key residues of the pocket. A sulfonyl group bonded with Asn194, one hydrogen-arene contact occurred between Ile130 and the nitrophenyl moiety, and hydrophobic interactions were observed between the aromatic ring and the Ala118 backbone of monomer A. Another sulfonyl group formed three polar bonds with Lys72, Tyr158 and Gln157 of monomer B. Compound 162530 exhibited seven contacts with key residues, but sulfonyl moieties were able to interact with pocket residues from two monomers A and B. One sulfonyl group bonded with the uridine-binding residues Asn194, Arg200 and Arg201 from monomer A, while two sulfonyl groups bonded with GlcNAc binding residues of Gln157 and Lys72 from monomer B. Compound 381868 showed three binding interactions, with Arg200, Gln157 and Met114. Compound 68858 displayed four bonds, such as hydroxy bonds, with Arg191 and hydroxy–ethoxy moiety showed one hydrogen–arene contact with Phe166 of monomer A. However, phosphoryl and ethoxy groups formed two bonds with Gln157 and His156 of monomer B. Compound 372529 exhibited five bonds, and the carboxylic group of butanoic acid showed one bond specifically implicated with product binding residue Met169 and Gln157. A sulfonamide group bonded with Phe66 and Ser150 of monomer A and one butanoic acid formed a bond with the His118 residue of monomer B. Compound 293892 showed eight bonds, with dihydropurine-2-6-dione showing two hydrogen–arene interactions with Phe166 and Gln196, hydroxy moiety bonds with Ser150 and Gly151, and a phenyl ring displaying an arene–hydrogen bond with Gly151 and Met169, and one polar bond with Met114. Compound 353465 displayed critical interactions with both monomers A and B, tetrahexoxa displayed two polar bonds via an oxygen atom with monomer B residues, such as Lys72 and Gln7, whereas the O atom of the tetraon showed two polar bonds with monomer A residues Asn194 and Arg201. Compound 366068 displayed three bonds, the O atom of azone bonding to Asn194 and Arg201 of monomer A, and the tricyclo tetracosane tetron forming two bonds with the GlcNAc binding pocket residues Lys72 and Gln157. Compound 321495 showed two bonds: an SH group of carbamimidothioic acid formed one bond with Arg200 of monomer A, and the S atom of the phenylmethanethiol group bonded with Asp70 from monomer B. Compound 7529 displayed five bonds, such as the 2, 5, dihydrofuran-2-one group forming two bonds with the uridine-binding residues Asn194 and Arg200 of monomer A, the 5OH of methyloxan forming key interactions with Asp70 and Lys71, and the 4OH and O groups of methyloxan bonding with Lys72 and Gln157 of monomer B. Thus, the assays of pharmacophore mapping, docking score, binding energy, binding affinity, binding pose and interactions with key residues of the binding pocket can lead to structurally diverse scaffolds that act as selective potent LpxA inhibitors (Figure 7). The molecular interaction analysis of these lead molecules can inhibit the UDP-GlcNAc pocket by competing with substrate. Subsequently, these compounds were evaluated for physicochemical, pharmacokinetic and ADME properties. The failure of drug accessing raises safety related concerns and is even more significant in the drug discovery pipeline, and actsas a guide to the medicinal chemist in creating drug design programs. And so, we utilized in silico approaches for identification of the ligand toxicophores and ADME properties.

### 3.7. Bioavailability and ADME Properties

**Figure 8:**
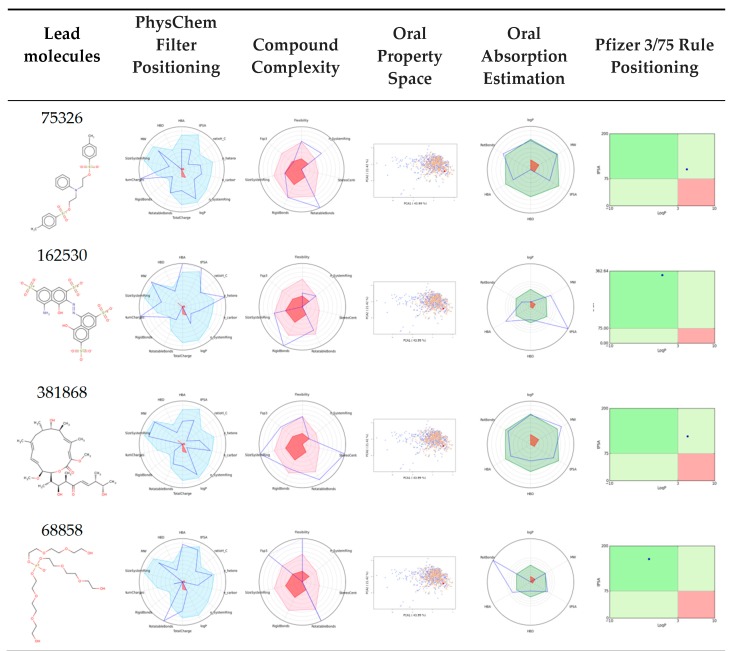

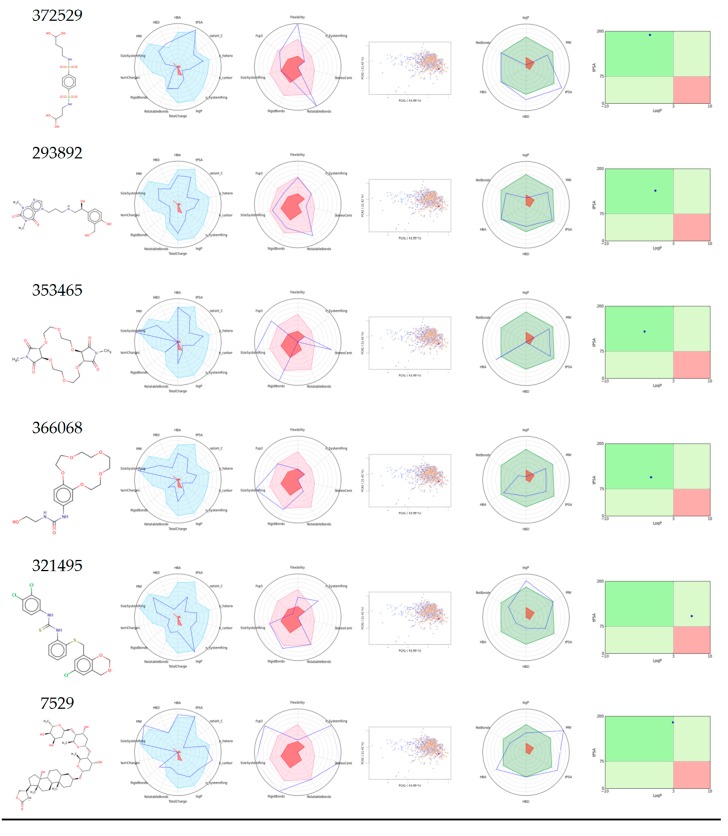
Drug likeness of leads was predicted using bioavailability radar. Pink depicts the optimal range of each property (Lipo: Lipophilicity, Size: Molecular weight, POLAR: Total Polar Surface Area, INSOLU: Insolubility, INSATU: Insaturation, FLEX: Flexibility).

Bioavailability of the lead molecules plays a major role in drug discovery, and molecular properties, pharmacodynamics, pharmacokinetics, the Lipinski rule, and toxicological properties are noteworthy to distinguish drug accessibility. However, pharmacodynamic findings have uncovered leads that have therapeutic potential by selectively targeting the active site of LpxA. The bioavailability for the leads was evaluated with the Swiss ADME and FAF-Drugs server. The bioavailability radar hat was generated which manifest promising drug likeness by using the Lipinski rule to predict lipophilicity (–0.7 to +0.5), size (150 g/mol > 500 g), polarity (20–130A), solubility (<6), flexibility (<9) and saturation (0.25–1) (Figure 8; Figure S3). The Lipinski rule of the leads computed that the molecular weight was found to be less than 500. The cLogP or partition coefficient played a critical role in the drug availability, and was determined to be less than five. Hydrogen bond acceptors were computed to be less than ten (≤10) and hydrogen bond donors to be less than five (≤5). Water solubility revealed that the absorption of drug molecules was projected to be soluble and moderately soluble. In addition, predictions of skin permeability, gastrointestinal absorption and blood brain barrier (BBB) permeability, as well as the interactions with different cytochrome p450 isoforms in drug elimination, were documented. Drug likeness mainly dealt with the molecular features that were compatible with absorption, distribution, metabolism and excretion in the body. Further, oral drug analysis was evaluated by estimating the Lipinski rule, Ghose (Amgen), Veber (GSK), Egan (Pharmacia) and Muegge (Bayer), which unambiguously determined it to be an oral drug (Table S2). Toxicity evaluations via mutagenic, tumorigenic, irritant and reproductive effects were determined to exert no adverse effect. A drug score was calculated based on the above four risk factors to identify all compounds displaying best drug score. Hence, these drug candidates might be promoted as promising antibacterial agents for the efficacious management of bacterial infections.

## 4. Conclusions

*P. aeruginosa* is a perilous multidrug-resistant Gram-negative bacterium, which drastically reduces the action of current drug potential through a variety of mechanisms and that often leads to therapeutic failure. LPS biosynthesis is an important mechanism in bacteria, and LpxA is a validated essential therapeutic target for design of potent antimicrobials against *P. aeruginosa*. In this study, crystal structures of LpxA from different bacterial pathogens were extracted and substrate binding cavity size, residue composition, hydrophilic and hydrophobic characteristics, and substrate binding position differences were analyzed. This information enabled us to rationally identify specific chemical groups to improve existing inhibitors and design new classes of selective inhibitors. We identified unique residue compositions at the active site, pocket volume and characteristic pocket of PaLpxA. Further, we created pharmacophore models using a complex-based approach and utilized crucial pharmacophoric regions implicated in key substrate-binding residues as a pharmacophore query to screen a heterogenous dataset to validate diverse scaffolds with potential pharmacophoric features. Subsequently, a combination of docking and Born integral volume energy studies was applied to determine binding energy and binding affinities of virtual hits to reveal 14 molecules as potential leads for PaLpxA inhibition. For these optimized potential leads, bioavailability properties were elucidated, revealing conspicuous remedial properties. However, to ensure their pharmacological properties, they must be assessed in further clinical stages. This study opens new observations for the generation of a rational drug discovery of LpxA antibacterial drugs with reference to clinical management of multidrug-resistant bacteria.

## Figures and Tables

**Table 1:** Crucial residues in the diversified LpxA UDP-GlcNAc binding pocket, root mean square deviation (RMSD) and pocket size from different orthologues.

Organism	PDB ID	Monomer A	Monomer B	RMSD (Å)	Pocket Size (Å)
		UDP Pocket	GlcNAc Pocket		
*P. aeruginosa*	5DEM	Arg200, Arg201, Gly197 Glu196, Asn194, Phe166, Ile148, Ile130, Leu112, Met114	Asp70, Leu71, Lys72, Ala138, Gly139, His121, His140, His156, Gln157, Tyr158		213
*E. coli*	2JF3	Arg204, Arg205, Glu201 Asn198, Met170, Ile152 Ile134, Leu116, Met118	Gln73, Asp74, Leu75, Lys76, His125, Gly143, His144, Gln161, Phe162	0.6	156
*B. fragilis*	4R37	Arg197, Arg198, Gly194, Ile193, Asn191, Ile199, Met163, Ile145, Ile127 Met111	Gln167, Asp68, Leu69, Lys70, His118, Gly136, Glu137, His153, Gln154, Phe155	0.8	123
*H. pylori*	IJ2Z	Arg197, His196, Asn194 Thr182, Met166, Asn148, Met130	Asp70, Leu71, Lys72, His121, Gly139, His140, Gln157, Phe158	0.6	208
*L. interrogans*	3HSQ	Arg199, Val195, Gly196, Asn193, Met163, Ile129	Gln168, Asp69, Leu70,, Gly71, His120, His130, Gly138, His155, Gln156, Phe157	0.8	205
*A. baumannii*	4E6Q	Asn197, Met185, Met169, Ile151, Ile133, Leu115, Met117	Gln172, Asp73, Leu74, Lys75, His124, Gly142, His143, His159, Gln160, Phe161	0.5	170
*B. thailandensis*	4EQY	Arg202, Gly198, Asn195, Ile183, Met167, Ile149, Val131, Met115	Gln70, Asp71, Met72, His122, Gly140, His141, His157, Gln158, Tyr159	0.6	145

**Table 2:** Binding interaction, distance and binding energy of peptide inhibitors with active site residues of PaLpxA.

Peptide Inhibitor	Binding Interactions Peptide	Protein	Distance (Å)	Binding Energy (Kcal/mol)
Peptide920	Trp13-----------Phe166 Met5-----------Asn194 Leu6-----------Arg200 Asp7-----------Arg200	monomer A	2.9 3.6 3.0 2.9 3.7 2.3 3.8	−263
	Ileu9-------------Lys72 Gly11-------------Lys72 Trp13------------His156	monomer B		
RJPXD33	Leu6------------Phe166 Met5-----------Met169	monomer A	2.9 2.4 3.5 3.5 3.4 3.3	−177
	Thr1------------His121 Thr1------------His118 Thr1------------Gln157 Asn2-------------Asp70	monomer B		

**Table 3:** Binding interaction, bond distances, bond angles, binding energies and binding affinities of the leads with PaLpxA.

Lead Molecules	Binding Interaction		Atoms Involved in Angle	Angle (∠)	Distance (Å)	Docking Score (S)	Binding Affinity (pKi)
	Protein	Ligand					
75326	Asn194----------O Ile130---------arene Ala138---------arene Lys72---------S=O Tyr158-------- S=O		OD2-OD1-H38 HE2-NE2-O27 HE2-NE2-O25 NE2-HE2-CN1 NE2-HE2-O26	26.5 49.2 40.4 24.4 40.6	3.4 3.2 3.3 3.6 3.9	−10.5	−6.7
162530	Asn194----------O=S Arg200---------O=S Arg201---------O-S Arg201---------O=S Lys72---------O=S Lys72---------O-S		NZ-HZ-O28 NE2-HE-O29 N1-N-C O27-N-C HE2-NE2-O25 NE2-HE2-CN1	64.6 53.6 22.9 95.0 49.2 79.8	3.0 3.7 3.1 3.1 4.3 3.3	−10.1	−6.9
381868	Arg200---------O Gln157---------O Met114--------OH		NZ-HZ-O31 NE2-HE-O32 N-N-C	30.5 35.4 21.8	3.1 3.0 4.0	−7.4	−7.9
68858	Phe166---------C Gln157---------O-C Gln157---------O-P His156---------O-P Arg191---------OH		NE2-HE-O27 ND1-CE1-H42 N-N-O N-O-C ND1-CE1-H42	69.2 84.1 29.6 19.9 80.3	2.9 3.9 3.2 3.5	−7.4	−7.1
372529	Phe166---------O=S Ser150---------HN Gln157---------C His118---------O Met169---------OH		NZ-HZ-O27 NE2-HE-N3 NE-HE-N3 NZ-HZ-O31 NE2-CD2-O28	33.8 35.3 90.8 60.7 84.1	3.1 3.3 3.9 3.1 4.0	−7.6	−7.6
293892	Phe166---------HN Ser150---------OH Glu196---------arene Gly151---------arene Gly151---------OH Met169---------arene Gly151---------C		CG-OD2-H46 NZ-HZ-O31 O-O-N O-N-C O-N-C OH-HH-O27 O-N-C	54.5 4.6 60.7 45.8 97.7 76.9 35.3	4.6 4.3 4.5 3.0 3.1 3.3 3.9	−7.3	−7.3
353465	Asn194----------O=C Arg201---------O=C Lys72-----------O Gln157---------O		F-N-C F-N-C C-C-O N-O-C	22.2 86.8 76.9 76.7	3.5 3.3 4.0 4.9	−8.9	−6.7
366068	Asn194----------OH Arg201---------OH Lys72---------O		HE2-NE2-O25 NE2-HE2-CN1 NE-HE-N3	45.8 97.7 76.9	3.3 3.9 3.1	−8.6	−6.4
321495	Arg200---------S Asp72---------S		NE2-HE-O29 C-C-O	60.7 45.8	3.7 3.1	−8.5	−6.6
7529	Asn194----------O Arg201---------O=C Lys72---------O Leu71---------OH Gln157---------O		NZ-HZ-O28 N1-N-C NE2-HE2-CN1 NZ-HZ-O31 OH-HH-O27	4.6 60.7 45.8 84.1 29.6	4.5 3.0 3.1 3.3 3.1	−8.5	−8.0

## Supplementary Materials

The supplementary materials are available online at https://www.mdpi.com/2218-273X/10/2/266/s1.
