# Supplementary material for: Structure-Based Virtual Screening of Pseudomonas aeruginosa LpxA Inhibitors using Pharmacophore-Based Approach
Source: Biomolecules. 2020 Feb 10;10(2):266. doi: 10.3390/biom10020266 (PMC7072397; doi:10.3390/biom10020266)
Supplement: Supplementary file 1 [file biomolecules-10-00266-s001.pdf]

biomolecules

MDPI

FIGURES

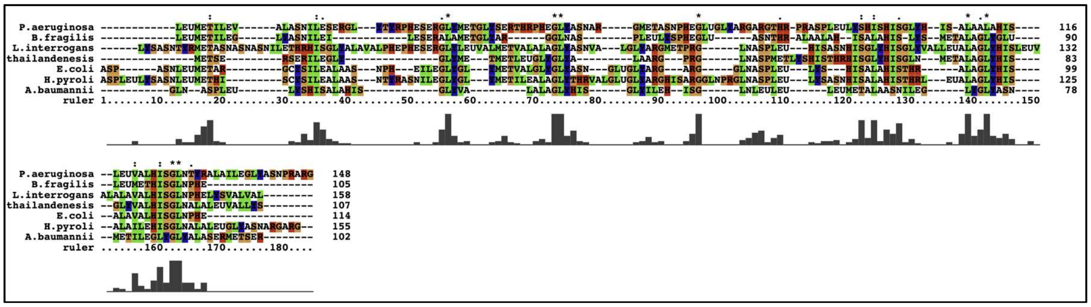

Figure S1. Multiple sequence alignment for UDP-GlcNAc pocket residues from different bacterial pathogens.

(**a**)

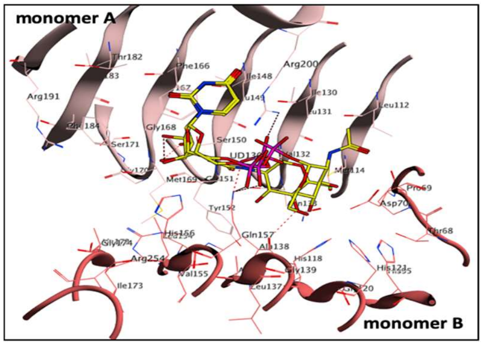

(**b**)

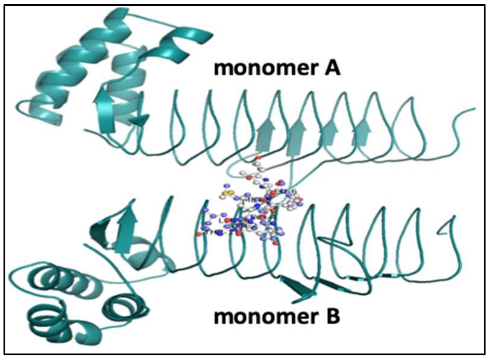

(**c**)

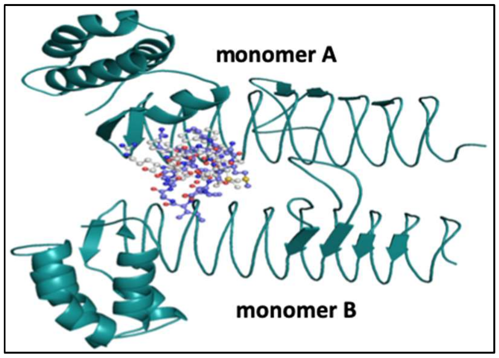

(**d**)

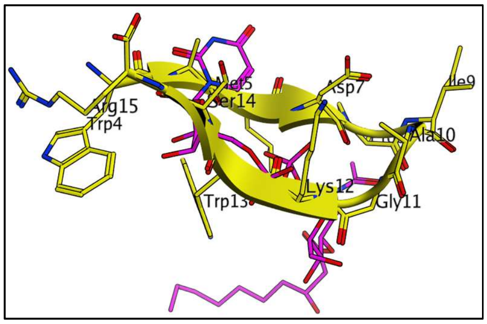

(**e**)

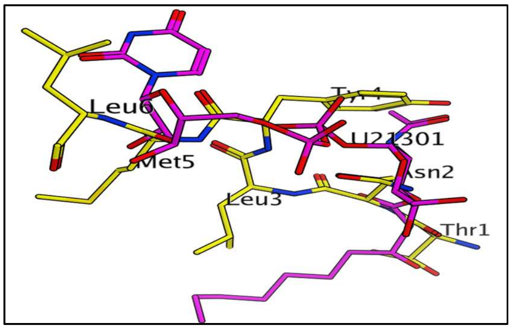

Figure S2. (**a**). 3D Overlay of docked pose and crystal pose of UDP-GlcNAc (yellow) within the active site of PaLpxA. (**b**). Overlays of RJDX33 docked pose (ball and stick, white) with crystal pose of RJDX33 (ball and sticks, blue) and peptide920 docked pose(ball and stick, white) with crystal pose of peptide920 (ball and sticks, blue). (**c**). docked peptide920 with crystal pose of peptide920 within the acyl-ACP site of *E. coli* LpxA. (**d**). Overlays of docked peptide920 (β-sheet with sticks by labelling (yellow), and (**e**). docked RJDX33 (sticks by labelling (yellow) with crystal conformation of UDP-3-O-(R-3-hydroxydecanoyl)-GlcNAc is depicted in sticks (magenta) of PaLpxA. **.**

75326

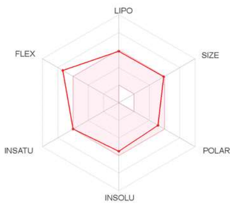

13340

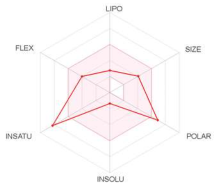

162530

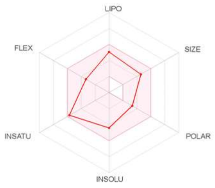

157638

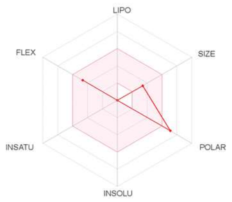

332180

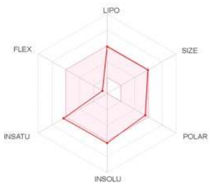

381868

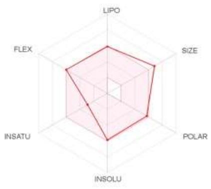

68858

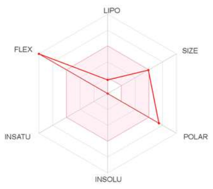

372529

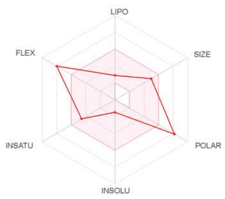

293892

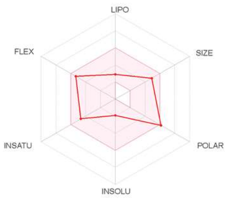

353465

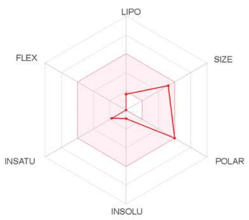

366068

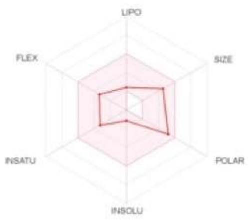

321495

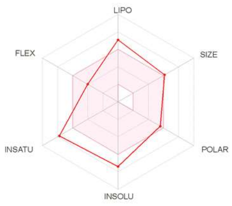

7529

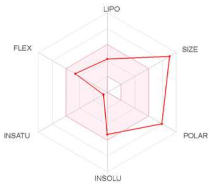

Figure S3. Drug-likeness of the PaLpxA was predicted using bioavailability radar. The pink is depicted the optimal range of each property (Lipo: Lipophilicity, Size: Molecular weight, POLAR: Total Polar Surface Area, INSOLU: Insolubility, INSATU: Insaturation, FLEX: Flexibility).

TABLES

Table S1. Percentage of identity of the pocket from LpxA orthologs.

S. No	Bacterial Pathogens	Identity (%)
1	B.fragilis vs. P.aeruginosa:	58.10
2	B.thailandenesis vs. P.aeruginosa	37.38
3	E.coli vs. P.aeruginosa	37.72
4	L.interrogans vs. P.aeruginosa	31.08
5	H.pyroli vs. P.aeruginosa	28.38
6	A.baumannii vs. P.aeruginosa	18.63

Table S2. Pocket size, propensity of the ligand binding (PLB), hydrophobicity (Hyd) and residues active pocket of LpxA orthologs. .

Bacterial Pathogens	Site	Size	PLB	Hyd	Side	Residues
P. aeruginosa	1	224	5.17	53	113	Monomer A<br>LEU112 MET114 ILE130 VAL132 ASN133 ILE148 SER150 GLY151 TYR152 PHE166 SER167 GLY168 MET169 GLY170 SER171 THR182 PHE184 GLY185 ASN186 ARG191 MET193 ASN194 PHE195 GLU196 GLY197 ARG200 ARG2013<br>Monomer B<br>THR68 PRO69 ASP70 LEU71 LYS72 HIS95 HIS118 GLY120 HIS121 ALA136 ALA138 HIS140 LEU154 VAL155 HIS156 GLN157 TYR158 ALA172 ILE173 GLY174 ASN186 PRO187 ARG254
E. coli	1	156	3.92	36	76	Monomer A<br>ASP114 ASN115 LEU116 MET118 ARG132 CYS133 ILE134 ALA136 ASN137 PHE150 ILE152 GLY154 GLY155 MET170 VAL171 GLY172 GLY173 ASN198 GLU200 GLY201 ARG204 ARG205<br>Monomer B<br>GLN73 ASP74 LEU75 LYS76 HIS122 ALA124 HIS125 THR140 ALA142 GLY143 HIS144 ALA158 VAL159 HIS160 GLN161 PHE162
B. fragilis	1	123	3.26	32	69	Monomer A<br>LEU109 MET111 ILE127 GLY129 ASN130 ILE145 ILE146 SER147 ALA148 MET163 GLY166 ARG197<br>Monomer B<br>GLN342 ASP343 LEU344 LYS345 PHE346 GLU349 ASN368 THR371 ALA372 ALA373 HIS390 ALA392 HIS393 LYS408 MET409 ALA410 GLY411 GLU412 LEU426 MET427 HIS428 GLN429 PHE430.

H. pylori	1	209	3.70	44	106	Monomer A<br>ASP85 LEU87 LYS110 ASN111 LEU112 MET114 HIS128 CYS129 ILE130 ALA132 ASN133 TYR146 ASN148 ILE149 GLY150 GLY151 MET166 ILE167 ALA168 GLY169 THR182 VAL183 GLU184 GLY185 ARG191 HIS196 ARG197 GLN200<br>Monomer B<br>PRO68 GLN69 ASP70 LEU71 LYS72 ASN95 HIS118 ALA120 HIS121 THR136 LEU137 ALA138 GLY139 HIS140 ALA154 ILE155 HIS156 GLN157 ALA172 LEU173 GLY174 ASN186 ARG187 ARG253
L. interrogans	1	205	5.23	52	105	Monomer A<br>LYS109 ASN110 TYR111 MET113 ASN115 ASN127 ASN128 ILE129 THR131 HIS132 GLY133 ALA134 VAL135 PHE145 PHE147 SER149 GLY150 LEU151 VAL152 MET165 VAL166 ALA167 GLY168 ASN193 VAL195 GLY196 ARG199<br>Monomer B<br>MET66 PRO67 GLN68 ASP69 LEU70 HIS95 ASN115 HIS117 GLY119 HIS120 GLY133 VAL135 LEU136 ALA137 GLY138 HIS139 LEU151 VAL152 ALA153 VAL154 HIS155 GLN156 PHE157 LYS171 VAL172 VAL173
A. baumannii	1	151	3.69	22	61	Monomer A<br>GLN72 ASP73 LEU74 LYS75 HIS121 ALA123 HIS124 GLY139 VAL140 ALA141 GLY142 HIS143 GLY157 ILE158 HIS159 GLN160 LEU175 LEU177<br>Monomer B<br>LEU115 MET117 ALA135 ASN136 ILE151 GLY153 GLY154 ASN155 MET169 ILE170 GLY171 GLY172 ALA173 SER174 MET185 SER187
B. thailandenesis	1	145	4.06	18	60	Monomer A<br>MET115 SER133 SER134 ILE149 GLY151 GLY152 MET153 MET167 LEU168 GLY169 GLY170 ALA171<br>Monomer B<br>ARG68 PRO69 GLN70 ASP71 MET72 LYS73 HIS96 THR99 HIS119 GLY121 HIS122 GLN137 MET138 ALA139 GLY140 HIS141 GLY155 VAL156 HIS157 GLN158 ALA173 LEU174 VAL175 LYS188

Table S3. Pysico-chemical properties, lipophilicity, solubility, pharmacokinetic, drug likeness and medicinal chemistry properties of the PaLpxA inhibtors.

Leads	Physicochemical Properties	Lipophilicity	Water Solubility	Pharmacokinetics	Drug likeness	Medicinal Chemistry
**75326**	Formula:C24H27NO6S2<br>Molecular weight: 489.60 g/mol<br>Num. heavy atoms: 33<br>Num. arom. heavy atoms: 18<br>Fraction Csp3: 0.25<br>Num. rotatable bonds: 11<br>Num. H-bond acceptors: 6<br>Num. H-bond donors: 0<br>Molar Refractivity: 127.59<br>TPSA: 106.74 Å²	Log $P_{o/w}$ (iLOGP): 3.84<br>Log $P_{o/w}$ (XLOGP3): 4.73<br>Log $P_{o/w}$ (WLOGP): 6.08<br>Log $P_{o/w}$ (MLOGP): 4.09<br>Log $P_{o/w}$ (SILICOS-IT):3.05<br>Consensus Log $P_{o/w}$: 4.36	Log *S* (ESOL): -5.53<br>Solubility: 1.43e-03 mg/ml ; 2.93e-06 mol/l<br>Class: Moderately soluble<br>Log *S* (Ali): -6.70<br>Solubility: 9.75e-05 mg/ml ; 1.99e-07 mol/l<br>Class: Poorly soluble<br>Log *S* (SILICOS-IT): -8.33<br>Solubility: 2.31e-06 mg/ml ; 4.72e-09 mol/l<br>Class:Poorly soluble	GI absorption: Low<br>BBB permeant: No<br>P-gp substrate: Yes<br>CYP1A2 inhibitor: No<br>CYP2C19 inhibitor:Yes<br>CYP2C9 inhibitor: Yes<br>CYP2D6 inhibitor: No<br>CYP3A4 inhibitor: No<br>Log K_p (skin permeation): -5.93 cm/s	Lipinski: Yes; 0 violation<br>Ghose: No; 2 violations: MW>480, WLOGP>5.6<br>Veber: No; 1 violation: Rotors>10<br>Egan: No; 1 violation: WLOGP>5.88<br>Muegge: Yes<br>Bioavailability Score: 0.55	PAINS: 0 alert<br>Brenk: 1 alert: sulfonic_acid_1<br>Lead likeness: No; 3 violations: MW>350, Rotors>7, XLOGP3>3.5<br>Synthetic accessibility: 3.35
**13340**	Formula:C9H11AsN2OS<br>Molecular weight: 334.18 g/mol<br>Num. heavy atoms: 18<br>Num. arom. heavy atoms:6<br>Fraction Csp3:0.11<br>Num. rotatable bonds:6<br>Num. H-bond acceptors:5<br>Num. H-bond donors:4<br>Molar Refractivity:66.96<br>TPSA: 155.02 Å²	Log $P_{o/w}$ (iLOGP):0.00<br>Log $P_{o/w}$ (XLOGP3):-0.66<br>Log $P_{o/w}$ (WLOGP):-1.19<br>Log $P_{o/w}$ (MLOGP):-1.69<br>Log $P_{o/w}$ (SILICOS-IT):-1.80<br>Consensus Log $P_{o/w}$: -1.07	Log *S* (ESOL): -1.35<br>Solubility 1.50e+01 mg/ml ; 4.50e-02 mol/l<br>Class:Very soluble<br>Log *S* (Ali): -2.12<br>Solubility:2.52e+00 mg/ml ; 7.56e-03 mol/l<br>Class: Soluble<br>Log *S* (SILICOS-IT):-1.49<br>Solubility:1.07e+01 mg/ml ; 3.21e-02 mol/l<br>Class:Soluble	GI absorption: Low<br>BBB permeant: No<br>P-gp substrate: No<br>CYP1A2 inhibitor: No<br>CYP2C19 inhibitor: No<br>CYP2C9 inhibitor: No<br>CYP2D6 inhibitor: No<br>CYP3A4 inhibitor: No<br>Log K_p (skin permeation): -8.81 cm/s	Lipinski:Yes; 0 violation<br>Ghose:No; 1 violation: WLOGP<-0.4<br>Veber:No; 1 violation: TPSA>140<br>Egan: No; 1 violation: TPSA>131.6<br>Muegge: No; 1 violation: TPSA>150<br>Bioavailability Score: 0.55	PAINS: 0 alert<br>Brenk: 2 alerts: heavy_metal, thioester<br>Leadlikeness: Yes<br>Synthetic accessibility: 2.92
**162530**	Formula:C20H15N3O14S4<br>Molecular weight:649.60 g/mol<br>Num. heavy atoms:41	Log $P_{o/w}$ (iLOGP):-1.43<br>Log $P_{o/w}$ (XLOGP3): 0.35<br>Log $P_{o/w}$ (WLOGP):6.72<br>Log $P_{o/w}$ (MLOGP):0.88	Log *S* (ESOL): -4.05<br>Solubility:5.75e-02 mg/ml ; 8.85e-05 mol/l<br>Class:Moderately soluble	GI absorption:Low<br>BBB permeant:No<br>P-gp substrate:Yes<br>CYP1A2 inhibitor:No	Lipinski: No; 3 violations: MW>500, NorO>10, NHorOH>5<br>Ghose: No; 3 violations: MW>480,	PAINS: 1 alert: azo_A<br>Brenk: 3 alerts: aniline, diazo_group, sulfonic_acid_2<br>Lead likeness:

	Num. arom. heavy atoms:20<br>Fraction Csp3:0.00<br>Num. rotatable bonds:6<br>Num. H-bond acceptors:16<br>Num. H-bond donors:7<br>Molar Refractivity:139.96<br>TPSA:342.20 Å²	Log $P_{o/w}$ (SILICOS-IT):-2.37<br>Consensus Log $P_{o/w}$:0.83	Log S (Ali):-7.10<br>Solubility:5.15e-05 mg/ml ; 7.93e-08 mol/l<br>Class:Poorly soluble<br>Log S (SILICOS-IT):-3.88<br>Solubility:8.47e-02 mg/ml ; 1.30e-04 mol/l<br>Class: Soluble	CYP2C19 inhibitor:No<br>CYP2C9 inhibitor:No<br>CYP2D6 inhibitor:No<br>CYP3A4 inhibitor:No<br>Log K_p (skin permeation):-10.01 cm/s	WLOGP>5.6, MR>130<br>Veber: No; 1 violation: TPSA>140<br>Egan: No; 2 violations: WLOGP>5.88, TPSA>131.6<br>Muegge: No; 4 violations: MW>600, TPSA>150, H-acc>10, H-don>5<br>Bioavailability Score:0.11	No; 1 violation: MW>350<br>Synthetic accessibility: 4.12
**157638**	Formula: C8H22N2O4P2<br>Molecular weight: 272.22 g/mol<br>Num. heavy atoms: 16<br>Num. arom. heavy atoms: 0<br>Fraction Csp3: 1.00<br>Num. rotatable bonds: 7<br>Num. H-bond acceptors: 4<br>Num. H-bond donors: 2<br>Molar Refractivity: 66.03<br>TPSA: 160.42 Å²	Log $P_{o/w}$ (iLOGP): 0.78<br>Log $P_{o/w}$ (XLOGP3): -6.47<br>Log $P_{o/w}$ (WLOGP): -0.60<br>Log $P_{o/w}$ (MLOGP): -8.08<br>Log $P_{o/w}$ (SILICOS-IT): 2.03<br>Consensus Log $P_{o/w}$:-2.47	Log S (ESOL): 3.01<br>Solubilit: 2.79e+05 mg/ml ; 1.02e+03 mol/l<br>Class: Highly soluble<br>Log S (Ali): 3.79<br>Solubility: 1.69e+06 mg/ml ; 6.22e+03 mol/l<br>Class: Highly soluble<br>Log S (SILICOS-IT): -1.32<br>Solubility: 1.29e+01 mg/ml ; 4.73e-02 mol/l<br>Class: Soluble	GI absorption: Low<br>BBB permeant: No<br>P-gp substrate: Yes<br>CYP1A2 inhibitor: No<br>CYP2C19 inhibitor: No<br>CYP2C9 inhibitor: No<br>CYP2D6 inhibitor: No<br>CYP3A4 inhibitor: No<br>Log Kp (skin permeation) -12.55 cm/s	Lipinski: Yes; 0 violation<br>Ghose: No; 1 violation: WLOGP<-0.4<br>Veber: No; 1 violation: TPSA>140<br>Egan: No; 1 violation: TPSA>131.6<br>Muegge: No; 2 violations: XLOGP3<-2, TPSA>150<br>Bioavailability Score: 0.55	PAINS: 0 alert<br>Brenk: 1 alert: phosphor<br>Lead likeness: Yes<br>Synthetic accessibility: 3.60
**332180**	Formula: C26H26N3O3S2<br>Molecular weight: 492.63 g/mol<br>Num. heavy atoms: 34<br>Num. arom. heavy atoms: 18<br>Fraction Csp3: 0.23<br>Num. rotatable bonds: 1<br>Num. H-bond acceptors: 4<br>Num. H-bond donors: 1<br>Molar Refractivity: 144.44	Log Po/w (iLOGP): -0.54<br>Log Po/w (XLOGP3): 4.86<br>Log Po/w (WLOGP): 4.25<br>Log Po/w (MLOGP): 2.20<br>Log Po/w (SILICOS-IT): 2.30<br>Consensus Log Po/w: 2.61	Log S (ESOL): -6.28<br>Solubility: 2.57e-04 mg/ml ; 5.23e-07 mol/l<br>Class: Poorly soluble<br>Log S (Ali): -7.06<br>Solubility: 4.26e-05 mg/ml ; 8.65e-08 mol/l<br>Class: Poorly soluble<br>Log S (SILICOS-IT): -7.62	GI absorption: High<br>BBB permeant: No<br>P-gp substrate: Yes<br>CYP1A2 inhibitor: Yes<br>CYP2C19 inhibitor: No<br>CYP2C9 inhibitor: No<br>CYP2D6 inhibitor: No<br>CYP3A4 inhibitor: Yes<br>Log K_p (skin permeation): -5.85 cm/s	Lipinski: Yes; 0 violation<br>Ghose: No; 2 violations: MW>480, MR>130<br>Veber: Yes<br>Egan: Yes<br>Muegge: Yes<br>Bioavailability Score: 0.55	PAINS: 0 alert<br>Brenk: 2 alerts: quaternary_nitrogen_1, thiol_2<br>Lead likeness: No; 2 violations: MW>350, XLOGP3>3.5<br>Synthetic accessibility: 6.16

	TPSA: 117.54 Å^2		Solubility: 1.19e-05 mg/ml ; 2.42e-08 mol/l<br>Class: Poorly soluble			
**381868**	Formula: C33H52O8<br>Molecular weight: 576.76 g/mol<br>Num. heavy atoms: 41<br>Num. arom. heavy atoms: 0<br>Fraction Csp3: 0.64<br>Num. rotatable bonds: 9<br>Num. H-bond acceptors: 8<br>Num. H-bond donors: 3<br>Molar Refractivity: 163.40<br>TPSA: 122.52 Å^2	Log $P_{o/w}$ (iLOGP): 4.77<br>Log $P_{o/w}$ (XLOGP3): 4.85<br>Log $P_{o/w}$ (WLOGP): 4.70<br>Log $P_{o/w}$ (MLOGP): 1.87<br>Log $P_{o/w}$ (SILICOS-IT): 4.16<br>Consensus Log $P_{o/w}$: 4.07	Log *S* (ESOL): -5.88<br>Solubility: 7.65e-04 mg/ml ; 1.33e-06 mol/l<br>Class: Moderately soluble<br>Log *S* (Ali): -7.16<br>Solubility: 4.02e-05 mg/ml ; 6.97e-08 mol/l<br>Class: Poorly soluble<br>Log *S* (SILICOS-IT): -2.96<br>Solubility: 6.35e-01 mg/ml ; 1.10e-03 mol/l<br>Class: Soluble	GI absorption: Low<br>BBB permeant: No<br>P-gp substrate: Yes<br>CYP1A2 inhibitor: No<br>CYP2C19 inhibitor: No<br>CYP2C9 inhibitor: No<br>CYP2D6 inhibitor: No<br>CYP3A4 inhibitor: Yes<br>Log K_p (skin permeation): -6.37 cm/s	Lipinski: Yes; 1 violation: MW>500<br>Ghose: No; 3 violations: MW>480, MR>130, #atoms>70<br>Veber: Yes<br>Egan: Yes<br>Muegge: Yes<br>Bioavailability Score: 0.55	PAINS: 0 alert<br>Brenk: 1 alert: michael_acceptor_1<br>Lead likeness: No; 3 violations: MW>350, Rotors>7, XLOGP3>3.5<br>Synthetic accessibility: 7.78
**68858**	Formula: C18H39O13P<br>Molecular weight: 494.47 g/mol<br>Num. heavy atoms: 32<br>Num. arom. heavy atoms:0<br>Fraction Csp3: 1.00<br>Num. rotatable bonds: 27<br>Num. H-bond acceptors: 13<br>Num. H-bond donors: 3<br>Molar Refractivity: 110.55<br>TPSA: 170.64 Å^2	Log $P_{o/w}$ (iLOGP): 4.64<br>Log $P_{o/w}$ (XLOGP3): -2.50<br>Log $P_{o/w}$ (WLOGP): -0.78<br>Log $P_{o/w}$ (MLOGP): -3.29<br>Log $P_{o/w}$ (SILICOS-IT): 1.64<br>Consensus Log $P_{o/w}$:-0.06	Log *S* (ESOL): 0.45<br>Solubility: 1.40e+03 mg/ml ; 2.83e+00 mol/l<br>Class: Highly soluble<br>Log *S* (Ali): -0.54<br>Solubility: 1.42e+02 mg/ml ; 2.88e-01 mol/l<br>Class: Very soluble<br>Log *S* (SILICOS-IT): -2.95<br>Solubility: 5.52e-01 mg/ml ; 1.12e-03 mol/l<br>Class: Soluble	GI absorption: Low<br>BBB permeant: No<br>P-gp substrate: Yes<br>CYP1A2 inhibitor: No<br>CYP2C19 inhibitor: No<br>CYP2C9 inhibitor: No<br>CYP2D6 inhibitor: No<br>CYP3A4 inhibitor: No<br>Log Kp (skin permeation) : -11.09 cm/s	Lipinski: Yes; 1 violation: NorO>10<br>Ghose: No; 3 violations: MW>480, WLOGP<-0.4, #atoms>70<br>Veber: No; 2 violations: Rotors>10, TPSA>140<br>Egan: No; 1 violation: TPSA>131.6<br>Muegge: No; 4 violations: XLOGP3<-2, TPSA>150, Rotors>15, H-acc>10<br>Bioavailability Score: 0.55	PAINS: 0 alert<br>Brenk: 1 alert: phosphor<br>Lead likeness: No; 2 violations: MW>350, Rotors>7<br>Synthetic accessibility: 5.39
**372529**	Formula: C14H22N2O8S2	Log $P_{o/w}$ (iLOGP): -26.78	Log *S* (ESOL): -1.49	GI absorption: Low	Lipinski: Yes; 1 violation: NHorOH>5	PAINS: 0 alert<br>Brenk: 0 alert

	Molecular weight: 410.46 g/mol<br>Num. heavy atoms: 26<br>Num. arom. heavy atoms: 6<br>Fraction Csp3: 0.43<br>Num. rotatable bonds: 12<br>Num. H-bond acceptors: 10<br>Num. H-bond donors: 6<br>Molar Refractivity:92.84<br>TPSA: 190.02 Å^2	Log $P_{o/w}$ (XLOGP3): -0.44<br>Log $P_{o/w}$ (WLOGP): 2.09<br>Log $P_{o/w}$ (MLOGP): -0.54<br>Log $P_{o/w}$ (SILICOS-IT): -2.91<br>Consensus Log $P_{o/w}$: -5.72	Solubility: 1.34e+01 mg/ml ; 3.26e-02 mol/l<br>Class: Very soluble<br>Log *S* (Ali): -3.09<br>Solubility: 3.37e-01 mg/ml ; 8.22e-04 mol/l<br>Class: Soluble<br>Log *S* (SILICOS-IT): -3.74<br>Solubility: 7.42e-02 mg/ml ; 1.81e-04 mol/l<br>Class: Soluble	BBB permeant: No<br>P-gp substrate: Yes<br>CYP1A2 inhibitor: No<br>CYP2C19 inhibitor: No<br>CYP2C9 inhibitor: No<br>CYP2D6 inhibitor: No<br>CYP3A4 inhibitor: No<br>Log K_P (skin permeation): -9.12 cm/s	Ghose: Yes<br>Veber: No; 2 violations: Rotors>10, TPSA>140<br>Egan: No; 1 violation: TPSA>131.6<br>Muegge: No; 2 violations: TPSA>150, H-don>5<br>Bioavailability Score: 0.11	Lead likeness: No; 2 violations: MW>350, Rotors>7<br>Synthetic accessibility: 2.94
**293892**	Formula: C19H27N5O5<br>Molecular weight: 405.45 g/mol<br>Num. heavy atoms: 29<br>Num. arom. heavy atoms: 12<br>Fraction Csp3: 0.47<br>Num. rotatable bonds: 8<br>Num. H-bond acceptors: 6<br>Num. H-bond donors: 5<br>Molar Refractivity: 115.78<br>TPSA: 131.99 Å^2	Log $P_{o/w}$ (iLOGP): 2.44<br>Log $P_{o/w}$ (XLOGP3): -0.13<br>Log $P_{o/w}$ (WLOGP): -2.24<br>Log $P_{o/w}$ (MLOGP): 0.22<br>Log $P_{o/w}$ (SILICOS-IT): 0.04<br>Consensus Log $P_{o/w}$: 0.06	Log S (ESOL): -2.05<br>Solubility: 3.61e+00 mg/ml ; 8.91e-03 mol/l<br>Class: Soluble<br>Log S (Ali): -2.19<br>Solubility: 2.63e+00 mg/ml ; 6.49e-03 mol/l<br>Class: Soluble<br>Log S (SILICOS-IT): -3.08<br>Solubility: 3.33e-01 mg/ml ; 8.22e-04 mol/l<br>Class: Soluble	GI absorption: Low<br>BBB permeant: No<br>P-gp substrate: Yes<br>CYP1A2 inhibitor: No<br>CYP2C19 inhibitor: No<br>CYP2C9 inhibitor: No<br>CYP2D6 inhibitor: No<br>CYP3A4 inhibitor: No<br>Log K_P (skin permeation) : -8.87 cm/s	Lipinski: Yes; 0 violation<br>Ghose: No; 1 violation: WLOGP<-0.4<br>Veber: Yes<br>Egan: No; 1 violation: TPSA>131.6<br>Muegge: Yes<br>Bioavailability Score: 0.55	PAINS: 0 alert<br>Brenk: 0 alert<br>Lead likeness: No; 2 violations: MW>350, Rotors>7<br>Synthetic accessibility: 4.15
**353465**	Formula: C18H26N2O10<br>Molecular weight: 430.41 g/mol<br>Num. heavy atoms: 30<br>Num. arom. heavy atoms: 0<br>Fraction Csp3: 0.78<br>Num. rotatable bonds: 0<br>Num. H-bond acceptors: 10<br>Num. H-bond donors: 0	Log $P_{o/w}$ (iLOGP): 2.11<br>Log $P_{o/w}$ (XLOGP3): -2.52<br>Log $P_{o/w}$ (WLOGP): -3.19<br>Log $P_{o/w}$ (MLOGP): -2.82<br>Log $P_{o/w}$ (SILICOS-IT): -1.22<br>Consensus Log $P_{o/w}$: -1.53	Log *S* (ESOL): -0.92<br>Solubility: 5.16e+01 mg/ml ; 1.20e-01 mol/l<br>Class: Very soluble<br>Log *S* (Ali): 0.33<br>Solubility: 9.22e+02 mg/ml ; 2.14e+00 mol/l<br>Class: Highly soluble	GI absorption: Low<br>BBB permeant: No<br>P-gp substrate: No<br>CYP1A2 inhibitor: No<br>CYP2C19 inhibitor: No<br>CYP2C9 inhibitor: No<br>CYP2D6 inhibitor: No<br>CYP3A4 inhibitor: No	Lipinski: Yes; 1 violation: NorO>10<br>Ghose: No; 1 violation: WLOGP<-0.4<br>Veber: Yes<br>Egan: Yes<br>Muegge: No; 1 violation: XLOGP3<-2<br>Bioavailability Score: 0.55	PAINS: 0 alert<br>Brenk: 1 alert: phthalimide<br>Lead likeness: No; 1 violation: MW>350<br>Synthetic accessibility: 4.76

	Molar Refractivity: 103.23<br>TPSA: 130.14 Å^2		Log *S* (SILICOS-IT): -0.74<br>Solubility: 7.80e+01 mg/ml ; 1.81e-01 mol/l<br>Class: Soluble	Log K_p (skin permeation): -10.71 cm/s		
**153371**	Formula: C19H18O6<br>Molecular weight: 342.34 g/mol<br>Num. heavy atoms: 25<br>Num. arom. heavy atoms: 14<br>Fraction Csp3: 0.21<br>Num. rotatable bonds: 5<br>Num. H-bond acceptors: 6<br>Num. H-bond donors: 4<br>Molar Refractivity: 95.03<br>TPSA: 107.22 Å^2	Log $P_{o/w}$ (iLOGP): 2.71<br>Log $P_{o/w}$ (XLOGP3): 4.55<br>Log $P_{o/w}$ (WLOGP): 3.31<br>Log $P_{o/w}$ (MLOGP): 1.91<br>Log $P_{o/w}$ (SILICOS-IT): 3.13<br>Consensus Log $P_{o/w}$: 3.12	Log *S* (ESOL): -4.91<br>Solubility: 4.18e-03 mg/ml ; 1.22e-05 mol/l<br>Class: Moderately soluble<br>Log *S* (Ali): -6.52<br>Solubility: 1.02e-04 mg/ml ; 2.99e-07 mol/l<br>Class: Poorly soluble<br>Log *S* (SILICOS-IT): -4.71<br>Solubility: 6.74e-03 mg/ml ; 1.97e-05 mol/l<br>Class: Moderately soluble	GI absorption: High<br>BBB permeant: No<br>P-gp substrate: No<br>CYP1A2 inhibitor: Yes<br>CYP2C19 inhibitor: No<br>CYP2C9 inhibitor: Yes<br>CYP2D6 inhibitor: Yes<br>CYP3A4 inhibitor: No<br>Log K_p (skin permeation): -5.16 cm/s	Lipinski: Yes; 0 violation<br>Ghose: Yes<br>Veber: Yes<br>Egan: Yes<br>Muegge: Yes<br>Bioavailability Score: 0.55	PAINS: 0 alert<br>Brenk: 2 alerts: hydroquinone, polycyclic_aromatic_hydrocarbon_2<br>Lead likeness: No; 1 violation: XLOGP3>3.5<br>Synthetic accessibility: 2.23
**345712**	Formula: C32H36O8<br>Molecular weight: 548.62 g/mol<br>Num. heavy atoms: 40<br>Num. arom. heavy atoms: 12<br>Fraction Csp3: 0.44<br>Num. rotatable bonds: 7<br>Num. H-bond acceptors: 8<br>Num. H-bond donors: 0<br>Molar Refractivity: 151.30<br>TPSA: 89.52 Å^2	Log Po/w (iLOGP): 4.68<br>Log Po/w (XLOGP3): 5.55<br>Log Po/w (WLOGP): 5.86<br>Log Po/w (MLOGP): 3.88<br>Log Po/w (SILICOS-IT): 7.06<br>Consensus Log Po/w: 5.41	Log S (ESOL): -6.50<br>Solubility: 1.74e-04 mg/ml ; 3.18e-07 mol/l<br>Class: Poorly soluble<br>Log S (Ali): -7.19<br>Solubility: 3.54e-05 mg/ml ; 6.45e-08 mol/l<br>Class: Poorly soluble<br>Log S (SILICOS-IT): -8.15<br>Solubility: 3.90e-06 mg/ml ; 7.11e-09 mol/l<br>Class: Poorly soluble	GI absorption: High<br>BBB permeant: No<br>P-gp substrate: Yes<br>CYP1A2 inhibitor: No<br>CYP2C19 inhibitor: No<br>CYP2C9 inhibitor: Yes<br>CYP2D6 inhibitor: No<br>CYP3A4 inhibitor: Yes<br>Log Kp (skin permeation): -5.71 cm/s	Lipinski: Yes; 1 violation: MW>500<br>Ghose: No; 4 violations: MW>480, WLOGP>5.6, MR>130, #atoms>70<br>Veber: Yes<br>Egan: Yes<br>Muegge: No; 1 violation: XLOGP3>5<br>Bioavailability Score: 0.55	PAINS: 0 alert<br>Brenk: 2 alerts: isolated_alkene, phenol_ester<br>Lead likeness: No; 2 violations: MW>350, XLOGP3>3.5<br>Synthetic accessibility: 5.38

**366068**	Formula: C17H26N2O7 Molecular weight: 370.40 g/mol Num. heavy atoms: 26 Num. arom. heavy atoms: 6 Fraction Csp3: 0.59 Num. rotatable bonds: 5 Num. H-bond acceptors: 7 Num. H-bond donors: 3 Molar Refractivity: 92.88 TPSA: 107.51 Å²	Log $P_{o/w}$ (iLOGP): 2.12 Log $P_{o/w}$ (XLOGP3): -1.27 Log $P_{o/w}$ (WLOGP): 0.43 Log $P_{o/w}$ (MLOGP): -0.78 Log $P_{o/w}$ (SILICOS-IT): 0.88 Consensus Log $P_{o/w}$: 0.27	Log S (ESOL): -1.18 Solubility: 2.46e+01 mg/ml ; 6.65e-02 mol/l Class: Very soluble Log S (Ali): -0.49 Solubility: 1.20e+02 mg/ml ; 3.23e-01 mol/l Class: Very soluble Log S (SILICOS-IT): -3.56 Solubility: 1.02e-01 mg/ml ; 2.77e-04 mol/l Class: Soluble	GI absorption: High BBB permeant: No P-gp substrate: Yes CYP1A2 inhibitor: No CYP2C19 inhibitor: No CYP2C9 inhibitor: No CYP2D6 inhibitor: No CYP3A4 inhibitor: No Log K_p (skin permeation): -9.46 cm/s	Lipinski: Yes; 0 violation Ghose: Yes Veber: Yes Egan: Yes Muegge: Yes Bioavailability Score: 0.55	PAINS: 0 alert Brenk: 0 alert Lead likeness: No; 1 violation: MW>350 Synthetic accessibility: 3.63
**321495**	Formula: C22H18Cl3N2O2S2 Molecular weight: 512.88 g/mol Num. heavy atoms: 31 Num. arom. heavy atoms: 18 Fraction Csp3: 0.14 Num. rotatable bonds: 6 Num. H-bond acceptors: 2 Num. H-bond donors: 2 Molar Refractivity: 135.04 TPSA: 118.44 Å²	Log $P_{o/w}$ (iLOGP): -0.18 Log $P_{o/w}$ (XLOGP3): 6.90 Log $P_{o/w}$ (WLOGP): 6.85 Log $P_{o/w}$ (MLOGP): 5.60 Log $P_{o/w}$ (SILICOS-IT): 5.65 Consensus Log $P_{o/w}$: 4.96	Log S (ESOL): -7.40 Solubility: 2.04e-05 mg/ml ; 3.98e-08 mol/l Class: Poorly soluble Log S (Ali): -9.20 Solubility: 3.25e-07 mg/ml ; 6.33e-10 mol/l Class: Poorly soluble Log S (SILICOS-IT): -9.89 Solubility: 6.58e-08 mg/ml ; 1.28e-10 mol/l Class: Poorly soluble	GI absorption: Low BBB permeant: No P-gp substrate: Yes CYP1A2 inhibitor: No CYP2C19 inhibitor: Yes CYP2C9 inhibitor: Yes CYP2D6 inhibitor: Yes CYP3A4 inhibitor: Yes Log K_p (skin permeation) : -4.53 cm/s	Lipinski: No; 2 violations: MW>500, MLOGP>4.15 Ghose: No; 3 violations: MW>480, WLOGP>5.6, MR>130 Veber: Yes Egan: No; 1 violation: WLOGP>5.88 Muegge: No; 1 violation: XLOGP3>5 Bioavailability Score: 0.17	PAINS: 0 alert Brenk: 3 alerts: imine_1, imine_2, thiol_2 Lead likeness: No; 2 violations: MW>350, XLOGP3>3.5 Synthetic accessibility: 3.90
**7529**	Formula: C41H64O13 Molecular weight: 764.94 g/mol Num. heavy atoms: 54 Num. arom. heavy atoms: 0	Log $P_{o/w}$ (iLOGP): 4.63 Log $P_{o/w}$ (XLOGP3): 1.85 Log $P_{o/w}$ (WLOGP): 3.25 Log $P_{o/w}$ (MLOGP): 1.12 Log $P_{o/w}$ (SILICOS-IT): 1.54	Log *S* (ESOL): -5.29 Solubility: 3.96e-03 mg/ml ; 5.17e-06 mol/l Class: Moderately soluble	GI absorption: Low BBB permeant: No P-gp substrate: Yes CYP1A2 inhibitor: No CYP2C19 inhibitor: No	Lipinski: No; 2 violations: MW>500, NorO>10 Ghose: No; 3 violations: MW>480, MR>130, #atoms>70	PAINS: 0 alert Brenk: 1 alert: saponine_derivative Lead likeness: No; 1 violation: MW>350 Synthetic accessibility: 8.74

	Fraction Csp3: 0.93<br>Num. rotatable bonds: 7<br>Num. H-bond acceptors: 13<br>Num. H-bond donors: 5<br>Molar Refractivity: 194.94<br>TPSA: 182.83 Å^2	Consensus Log $P_{o/w}$: 2.48	Log *S* (Ali): -5.31<br>Solubility: 3.74e-03 mg/ml ; 4.89e-06 mol/l<br>Class: Moderately soluble<br>Log *S* (SILICOS-IT): -2.26<br>Solubility: 4.23e+00 mg/ml ; 5.53e-03 mol/l<br>Class: Soluble	CYP2C9 inhibitor: No<br>CYP2D6 inhibitor: No<br>CYP3A4 inhibitor: No<br>Log K_p (skin permeation): -9.65 cm/s	Veber: No; 1 violation: TPSA>140<br>Egan: No; 1 violation: TPSA>131.6<br>Muegge: No; 4 violations: MW>600, TPSA>150, #rings>7, H-acc>10<br>Bioavailability Score: 0.17

Table S4. SMILES and IUPAC names of PaLpxA inhibitors.

Leads	SMILES	IUPAC Name
75326	CC1=CC=C(C=C1)[S](=O)(=O)OCCN(CCO[S](=O)(=O)C2=CC=C(C)C=C2)C3=CC=CC=C3	2-({2-[(4methylbenzenesulfonyl)oxy]ethyl}(phenyl)amino)ethyl 4-methylbenzene-1-sulfonate
13340	NC(=O)SCC(=O)NC1=CC=C(C=C1)[As](O)(O)=O	4-({1-[5-(4hydroxyphenyl)-2,5-dihydro-1H-pyrazole-3-carbonyl]pyrrolidin-3-yl}methyl) benzoic acid
162530	NC1=C2C(=C(N=NC3=C4C(=CC(=CC4=CC(=C3)[S](O)(=O)=O)[S](O)(=O)=O)O)C(=CC2=CC(=C1)[S](O)(=O)=O)[S](O)(=O)=O)O	8-[(1E)-2-[1-hydroxy-8-imino-3,6-bis(trioxo-lambda7sulfanyl) 3naphthalen-2-yl]diazen-1-yl]-3,6-bis(trioxo-lambda7-sulfanyl)naphthalen-1-ol
157638	CC(C)([NH2+]CC[NH2+]C(C)(C)[PH]([O-])=O)[PH]([O-])=O	(2-phosphopropan-2-yl)({2-[(2-phosphopropan-2-yl)amino]ethyl})amine
332180	[H][n]1-c2c([H])c([H])c([H])c(c2[H])S(=O)(=O)N2C([H])([H])C([H])([H])C([H])([H])C([H])([H])[C@@]2([H])C([H])([H])c2c([H])c([H])c(c([H])c2[H])C(=O)c(c1S):n1c([H])c([H])c([H])c([H])c1[H]	1-[(16Z)-9,9,18-trioxo-16-sulfanyl-9lambda6-thia-8,15-diazatetracyclo[17.2.2.1^{10,14}.0^{3,8}]tetracosa-1(21),10,12,14(24),16,19,22-heptaen-17-yl]-1lambda5-pyridin-1-ylium
381868	CO[C@H]1\C=C\C=C(C)\C[C@H](C)[C@H](O)[C@@H](C)\C=C(/C)\C=C(OC)\C(=O)O[C@H]1[C@@H](C)[C@H](O)[C@@H](C)C(=O)\C=C\[C@H](C)[C@H](C)O	(3Z,5E,7S,8S,9S,11E,13E,15S,16S)-16-[(2S,3S,4R,6E,8S,9S)-3,9-dihydroxy-4,8-dimethyl-5-oxodec-6-en-2-yl]-8-hydroxy-3,15-dimethoxy-5,7,9,11-tetramethyl-1-oxacyclohexadeca-3,5,11,13-tetraen-2-one
68858	OCCOCCOCCO[P](=O)(OCCOCCOCCO)OCCOCCOCCO	2-[2-(2-{[bis({2-[2-(2 hydroxyethoxy)ethoxy]ethoxy})phosphoryl]oxy}ethoxy)ethoxy]ethan-1-ol
372529	OC(=[OH])CCCNS(=O)(=O)c1ccc(cc1)S(=O)(=O)NCCCC(=[OH])O	4-{4-[(3-carboxypropyl)sulfamoyl]benzenesulfonamido}butanoic acid
293892	CN1C2=C(N(CCCNC[C@@H](O)C3=CC(CO)=C(O)C=C3)C=N2)C(=O)N(C)C1=O	7-(3-{[(2S)-2-hydroxy-2-[4-hydroxy-3-(hydroxymethyl)phenyl]ethyl]amino}propyl)-1,3-dimethyl-1,2-dihydropurine-2,6-dione
353465	CN1C(=O)C2OCCOCCOC3C(OCCOCCOC2C1=O)C(=O)N(C)C3=O	(1R,9R,13R,21R)-11,23-dimethyl-2,5,8,14,17,20-hexaoxa-11,23-diazatricyclo[19.3.0.0^{9,13}]tetracosane-10,12,22,24-tetrone
366068	[H]OC([H])([H])C([H])([H])N([H])C(=O)N([H])c1c([H])c([H])c2OC([H])([H])C([H])([H])OC([H])([H])C([H])([H])OC([H])([H])C([H])([H])OC([H])([H])C([H])([H])Oc2c1[H]	3-(2-hydroxyethyl)-1-(2,3,5,6,8,9,11,12-octahydro-1,4,7,10,13-benzopentaoxacyclopentadecin-15-yl)urea

32149 5	SC(NC1=CC(Cl)=C(Cl)C =C1)=NC1=C(SCC2=C3 OCOCC3=CC(Cl)=C2)C =CC=C1	N'-(2-{[(6-chloro-2,4-dihydro-1,3-benzodioxin-8yl)methyl]sulfanyl}phenyl)-N-(3,4-dichlorophenyl)carbamimidothioic acid
7529	O=C1OCC(=C1)[C@H]1 CC[C@@]2([C@]1(C)CC[C@H]1[C@H]2CC[C@@ H]2[C@]1(C)CC[C@@H](C2)O[C@H]1C[C@@H](O)[C@H]([C@@H](O1)C) O[C@H]1C[C@@H](O)[C@H]([C@@H](O1)C)O[C@H]1C[C@@H](O)[C@ H]([C@@H](O1)C)O)O	4[(1R,3aR,3bR,5aS,7S,9aS,9bS,11aR)-7-{[(2R,4R,5R,6S)-5-{[(2S,4R,5R,6S)-5-{[(2S,4R,5R,6S)-4,5-dihydroxy-6-methyloxan-2-yl]oxy}-4-hydroxy-6-methyloxan-2-yl]oxy}-4-hydroxy-6-methyloxan-2-yl]oxy}-3a-hydroxy-9a,11a-dimethyl-hexadecahydro-1H-cyclopenta[a]phenanthren-1-yl]-2,5-dihydrofuran-2-one.
